# Metabolomic Profiles and Anti-Herpes Simplex Virus (Wild-Type and Drug-Resistant) Properties of Water-Based Extracts of *Lentinula edodes*, *Hypsizygus marmoreus* and *Pleurotus eryngii*

**DOI:** 10.3390/molecules31122091

**Published:** 2026-06-14

**Authors:** Chaleampol Loymunkong, Chamsai Pientong, Tipaya Ekalaksananan, Yaovapa Aramsirirujiwet, Jureeporn Chuerduangphui

**Affiliations:** 1 Department of Microbiology, Faculty of Science, Kasetsart University, Bangkok 10900, Thailand; chaleampol.lo@ku.th (C.L.); fsciypt@ku.ac.th (Y.A.); 2 Department of Microbiology, Faculty of Medicine, Khon Kaen University, Khon Kaen 40002, Thailand; chapie@kku.ac.th (C.P.); tipeka@kku.ac.th (T.E.); 3 HPV & EBV and Carcinogenesis Research Group, Khon Kaen University, Khon Kaen 40002, Thailand

**Keywords:** *Lentinula edodes*, *Hypsizygus marmoreus*, *Pleurotus eryngii*, herpes simplex virus, molecular docking, metabolite, network pharmacology, ESI-QTOF-MS, bioactive compound

## Abstract

Herpes simplex virus type 1 (HSV-1) remains a significant pathogen, particularly in immunocompromised patients. The emergence of drug-resistant strains necessitates alternative therapeutic agents. *Lentinula edodes* (LE), *Hypsizygus marmoreus*, and *Pleurotus eryngii* are edible mushrooms with recognized medicinal properties. However, their effects on drug-resistant HSV-1 remain unclear. This study characterized metabolites from high-temperature/high-pressure (121 °C) water extracts of fresh and dried fruiting bodies and evaluated anti-HSV-1 activities using in vitro and in silico approaches. Metabolic profiles were analyzed by electrospray ionization–quadrupole time-of-flight mass spectrometry. Antiviral activity against HSV-1 KOS (wild-type) and HSV-1 dxpiii (drug-resistant) strains was assessed by plaque assays and qPCR. Molecular docking and network pharmacology were performed on candidate compounds. LE extract from dried mushroom tended to show the highest levels of selected major bioactive constituents, along with greater antioxidant activities. All extracts significantly inhibited viral infection and gene expression in both strains. LE extract from dried mushroom modulated the expression of *NFKB1* and *IL6*. Molecular docking analysis revealed that eritanidine showed a predicted binding affinity to HSV-1 DNA polymerase (−7.95 kcal/mol). Additionally, eritanidine, 5′-methylthioadenosine, and 3-indoleacrylic acid were predicted to interact with TNF and MAPK1. Several compounds also demonstrated favorable drug-likeness properties. Overall, these mushroom extracts are promising natural sources of antiviral agents against HSV-1, including drug-resistant variants.

## 1. Introduction

Herpes simplex virus type 1 (HSV-1) infection is a major global health burden, with more than 3 billion individuals estimated to be HSV-1-positive [1]. HSV-1 is a contact-transmissible pathogen that causes cutaneous and mucosal herpetic lesions in humans and may lead to severe complications, including keratitis and life-threatening disseminated disease or encephalitis [2]. Following primary infection, HSV-1 establishes latency in neuronal ganglia, which is typically characterized by lifelong periodic reactivations [3]. Long-term use of acyclovir (ACV) in HSV treatment, especially in immunocompromised patients, is associated with the development of drug resistance [2,4,5,6]. Frequent recurrence of HSV lesions can lead to the emergence of ACV-resistant strains, reported in approximately 1.95–6.4% of cases [6,7]. Therefore, new agents are urgently needed to combat HSV-1 infections, especially to overcome drug resistance and the limitations of standard ACV. While ACV primarily targets viral DNA polymerase to inhibit replication in infected cells, bioactive compounds from mushrooms, such as polysaccharides and polyphenols, have been reported to exhibit antiviral properties through multiple mechanisms, including inhibition of viral entry and modulation of host immune responses [8]. Consequently, combining ACV with mushroom extracts may represent a multi-targeted approach. This combination has the potential to enhance antiviral efficacy, reduce the required therapeutic doses, and limit the risk of resistance, warranting further investigation of possible synergistic effects against HSV-1.

*Lentinula edodes* (LE), *Hypsizygus marmoreus* (HM), and *Pleurotus eryngii* (PE) are edible mushrooms widely cultivated and utilized in Asian traditional medicine, particularly in China, Japan, and Korea [9,10,11]. LE enhanced immunity in humans, and promoted longevity in *Drosophila melanogaster* [12,13]. Similarly, PE has been documented in traditional medicine for stimulating immune function, supporting wound healing, and promoting joint and muscle relaxation [12,14,15]. HM also exhibits several medicinal properties, including anti-tumor, anti-aging, and antimicrobial activities [16,17,18]. Although the metabolic profiles of these three edible mushrooms have been individually reported, no study has conducted a comparative metabolomic analysis, particularly using extracts prepared under high-temperature (121 °C) and high-pressure (15 psi) conditions, which may be relevant to their biological activities and suggest favorable in vitro safety profiles [19,20,21]. In addition, the inhibitory effects of LE against drug-susceptible strains have been reported; however, their activities against drug-resistant HSV strains have not been well documented [22,23]. Moreover, antiviral activities of HM and LE against either drug-susceptible or drug-resistant HSV-1 strains remain limited [24].

Numerous studies have identified various bioactive compounds in edible mushrooms, including polysaccharides, terpenoids, phenolic acids, and sterols, all of which contribute to their pharmacological properties, including anticancer, immunomodulatory, antioxidant, antimicrobial, and antiviral activities [9,10,11]. Despite extensive ethnopharmacological use and accumulating evidence of biological activity, the specific metabolites and molecular mechanisms underlying the antiviral properties of these mushrooms, particularly their interactions with host cellular pathways exploited by viruses, remain poorly understood. A previous study demonstrated that *Peganum harmala* L. leaf extract exerted anti-HSV-1 activity, with network pharmacology analysis identifying MAPK1, SRC, EGFR, and JAK1 as key putative HSV-1-associated genes, which were highly enriched in the MAPK, PI3K-Akt, and JAK-STAT signaling pathways and were primarily associated with the mechanisms of action of its bioactive compounds [25]. Correspondingly, other studies have reported integrated network pharmacology analyses of the Chinese herbal prescription JieZe-1 against HSV-2, revealing 36 cellular hub targets, including EGFR, IL1B, IL6, MAPK1, SRC, and STAT [26]. In addition, chrysin (5,7-dihydroxyflavone), a compound found in *Pleurotus ostreatus*, was shown to share common targets such as EGFR and SRC in its activity against HSV-2 [27]. Unfortunately, an integrating network pharmacology and molecular docking of LE, HM and PE-derived compounds against HSV and cellular proteins has not been elucidated.

To address these gaps, the present study compares the bioactive ingredients and untargeted metabolite profiles of fresh (LE_F, HM_F, and PE_F) and dried (LE_D, HM_D, and PE_D) extracts prepared under high-temperature/high-pressure conditions and evaluates their inhibitory effects against both drug-susceptible and drug-resistant HSV-1. By integrating analytical and computational approaches, this work highlights candidate compounds from these edible mushrooms for future development as health-promoting products and natural antiviral agents.

## 2. Results and Discussion

### 2.1. Bioactive Compound Profiles of Fresh and Dried Mushroom Extracts

To assess the high-temperature/high-pressure extract method, the final yield of the lyophilized powder was calculated relative to the initial 200 g of fresh mushroom. The resulting lyophilized powder yields 9.65, 9.40, and 9.25 mg/g for LE_F, HM_F, and PE_F, respectively, and 10.45, 9.75, and 9.45 mg/g for LE_D, HM_D, and PE_D, respectively. Comparative analysis of fresh and dried extracts of three edible mushrooms, including LE, HM, and PE revealed notable differences in their bioactive profiles (Table 1). Drying tended to increase total polysaccharides and β-glucans, with LE_D showing the highest levels (138.35 ± 3.29 mg/g and 9.54% ± 0.23% *w*/*w*, respectively). TPC was similar across all extracts (0.01–0.06 mg/g), while terpenoids were most abundant in LE_F (6.0 ± 0.40 mg/g) and LE_D (6.6 ± 0.70 mg/g). Protein content was consistently higher in dried extracts, with LE_D reaching 17.5 ± 0.64 mg/g. LE_D also exhibited the strongest antioxidant capacity (DPPH 0.99 ± 0.01 mg GAE/g; FRAP 3.11 ± 0.00 mg GAE/g) (Table 1). Overall, drying may contribute to improved nutritional properties and bioactive compound levels, supporting the traditional use of dried mushrooms and their potential as functional antiviral resources.

The extraction of LE, HM, and PE has been performed using various techniques and solvents. Water-based extraction offers several advantages, including ease of operation, low cost, high safety, and the absence of residual solvent contamination [28]. However, the extraction efficiency of this method may be limited, and it is primarily used to isolate water-soluble components such as phenolic compounds, lipids and fatty acids, polysaccharides, proteins, vitamins, and minerals [29]. High-temperature and high-pressure water extraction at 121 °C and 15 psi for 1 h was employed in this study to obtain bioactive compounds from the three mushroom species. Under these conditions, water can effectively penetrate the mushroom cell walls, thereby facilitating the dissolution of soluble polysaccharides, including β-glucans, as well as secondary metabolites [30,31]. However, such conditions may also lead to the degradation of certain bioactive compounds. Yu et al. [21] investigated PE from fresh mushrooms subjected to air frying at 180 °C for 30 min, boiling for at least 90 min, stir-frying for 8 min, and steaming for 15 min. The metabolite profiles obtained from all four cooking methods were clearly distinguishable from those of the raw material, whereas only minor differences were observed among the four processed samples. Vetvicka et al. [32] reported that a dried PE extract obtained by hot water extraction at 121 °C for 30 min in an autoclave, followed by ethanol precipitation, yielded 43.70% ± 0.89% β-glucan and 0.90% ± 0.01% protein. In contrast, our study observed slightly lower yields, ranging from 8.16% ± 0.14% to 8.68% ± 0.04% for β-glucans and from 0.64% ± 0.13% to 0.91% ± 0.34% for proteins (equivalent to 6.4 ± 1.32 to 9.1 ± 3.48 mg/g) [32]. Udchumpisai et al. [33] demonstrated that crude polysaccharide extracts derived from boiled and dried fruiting bodies of LE contained total carbohydrates of 411.98 ± 35.95 mg/g and proteins of 275.42 ± 9.67 mg/g, which were markedly higher than those observed in our study (126.55 ± 0.22 to 138.35 ± 3.29 mg/g and 14.60 ± 2.03 to 17.50 ± 0.64 mg/g, respectively). In the same study, crude extracts obtained using 95% ethanol yielded 83.02 ± 4.37 and 77.14 ± 9.05 mg/mg of carbohydrates and proteins, respectively [33]. Kozarski et al. reported that crude hot-water polysaccharide extracts from LE contained 15.0 ± 2.0 mg/g (1.5 ± 0.2 g/100 g) of total polysaccharides, 0.8 ± 0.1 g/100 g of β-glucans, 0.20 ± 0.00 mg/g (0.02 ± 0.00 g/100 g) of TPC, less than 0.1 mg extract/g of DPPH radical scavenging activity, and 8.26 ± 0.03 mg extract/mL of chelating ability on ferrous ion activity [34]. Kała et al. [35] elucidated β-glucan yields ranging from 28.2% ± 1.1% to 46.8% ± 1.4% (28.2 ± 1.1 to 46.8 ± 1.4 g/100 g). Notably, the DPPH radical scavenging activity of their methanol-based HM extracts was substantially higher than that observed in our study [35]. Unlike many studies that preferentially precipitate carbohydrates or β-glucans using ethanol, our extraction method omitted this step to retain not only polysaccharides but also other classes of chemical compounds that may collectively contribute to anti-HSV activity. Consequently, this lack of precipitation could explain the lower relative yields of polysaccharides and β-glucans observed in our study compared to those focusing solely on carbohydrate purification. Importantly, despite methodological differences, the profile of bioactive compounds obtained in our study was broadly comparable to those reported in previous studies.

### 2.2. Untargeted Metabolites Analysis of the Extracts

The chromatograms of the electrospray ionization–quadrupole time-of-flight mass spectrometry (ESI-QTOF-MS) of six mushroom extracts are shown in Figure S1. A total of 2845 and 951 metabolites were putatively annotated in positive- and negative-ion modes, respectively, based on MS/MS spectral matching, retention time, and accurate mass using the Bruker MetaboBase Personal Library 2.0 and the MassBank of North America (MONA) database (Table 2 and Table 3, File S1). The annotated metabolites mainly belonged to amino acid derivatives, alkaloids, indoles, carnitine derivatives, sphingolipids, and organic compounds. Drying markedly influenced the metabolite profiles. In positive-ion mode, eritanidine (ETD) and γ-glutamylleucine (GGL) were more abundant in LE_F, while 2-methylbutyroylcarnitine (MBC) and p-tolyldiethanolamine were relatively higher in LE_D (Table 2). In HM, indoline and 3-indoleacrylic acid (IAL) were enriched in HM_F, whereas HM_D contained relatively higher levels of *N*-acetyl-2-phenylethylamine and phytosphingosine. In PE, amino acid derivatives predominated in PE_F, while PE_D exhibited an increase in aromatic amines and lipid-like metabolites, including tetramethylpyrazine. In negative-ion mode, the dominant metabolites were mainly organic acids and sugar alcohols. Malic acid was the most abundant compound found in LE_F, LE_D, HM_F and PE_F (Table 3), while phenyllactic acid (PLA) was relatively more abundant in HM_D and PE_D. Overall, fresh and dried samples displayed distinct metabolic signatures, demonstrating that drying may influence metabolite composition and relative abundance.

### 2.3. Metabolomic Characterization of the Extracts

**Figure 1:**
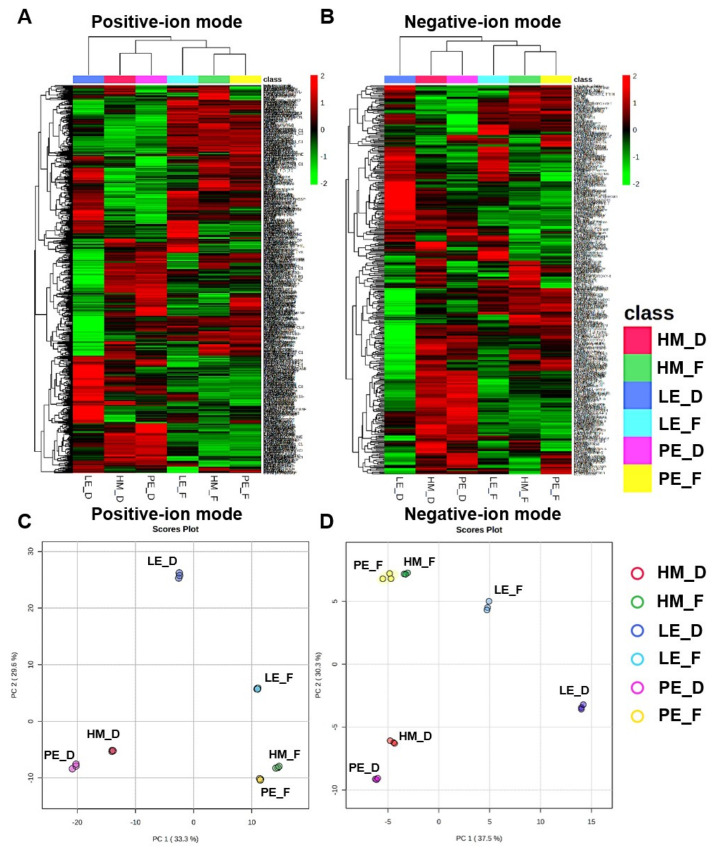
Untargeted metabolomic profiles of LE, HM, and PE extracts analysis by ESI-QTOF-MS in positive- and negative-ion modes. (**A**,**B**) Heat maps show overall metabolite profiles of six extracts in (**A**) positive- and (**B**) negative-ion modes. Red and green colors represent high and low average intensity, respectively. (**C**,**D**) PCA score plots are analyzed to elucidate the different metabolites of six extracts in (**C**) positive- and (**D**) negative-ion modes.

**Figure 2:**
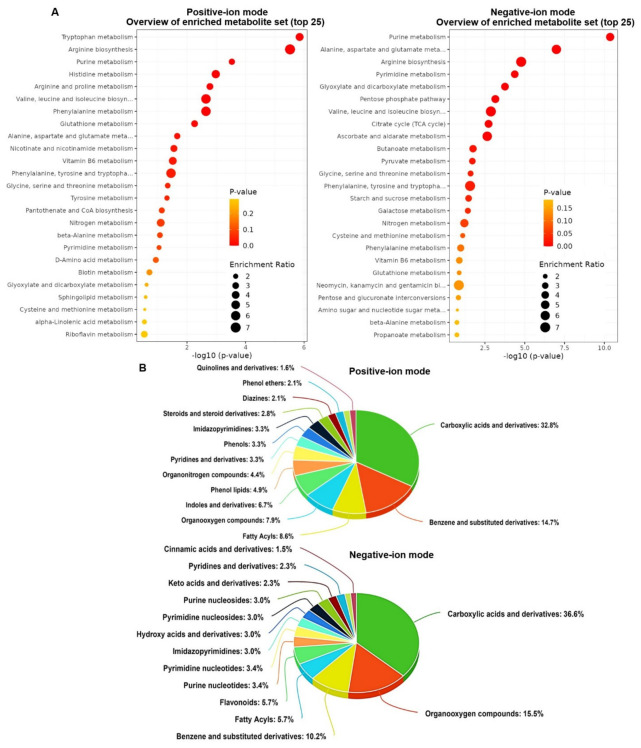
Key biological pathways and chemical classification of detected metabolites of all extracts in positive- and negative-ion modes. (**A**) The top 25 enriched metabolite sets were identified across the six extracts. (**B**) Pie chart illustrates the chemical classification of detected metabolites in the six extracts.

Mass spectrometry profiling of the six extracts revealed clear species-specific clustering. Hierarchical heatmaps of the overall metabolite profiles and the top 50 significant features (FDR < 0.05), and principle component analysis (PCA) plots showed distinct separation of LE_F, LE_D, HM_F, HM_D, PE_F and PE_D, which clustered closely, indicating similar metabolite compositions (Figure 1 and Figure S2). PCA explained 33.3% and 29.6% of total variance in positive-ion mode, and 37.5% and 30.3% in negative-ion mode. Metabolite set enrichment analysis highlighted 25 key biological pathways associated with these extracts (Figure 2A). Chemical classification indicated that carboxylic acids and derivatives predominated (33.8% in positive and 38.6% in negative modes), followed by benzene derivatives and fatty acyls or organooxygen compounds (Figure 2B). Overall, the metabolomic profiling demonstrates diverse and systematic bioactive compositions, with LE showing distinct metabolic features compared to HM and PE. The abundance of carboxylic acids, benzene derivatives, and organooxygen compounds supports the ethnopharmacological and functional potential of these edible mushrooms.

### 2.4. Antiviral Activity of Mushroom Extracts Against HSV-1_WT and HSV-1_Dxpiii

#### 2.4.1. Pre-Entry Step

**Figure 3:**
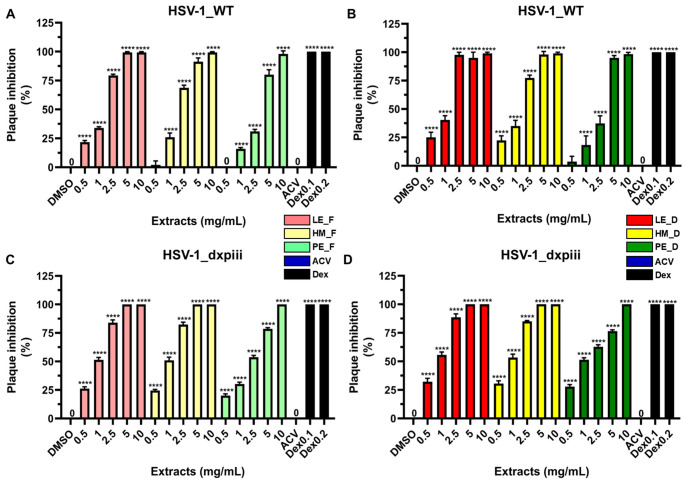
Antiviral effect of LE_F, LE_D, HM_F, HM_D, PE_F and PE_D extracts on plaque reduction in HSV-1_WT and HSV-1_dxpiii infections at the pre-entry step. (**A**,**C**) Fresh and (**B**,**D**) dried extracts were treated in (**A**,**B**) HSV-1_WT and (**C**,**D**) HSV-1_dxpiii-infected cells at the pre-entry step. ACV (1 mg/mL), Dex (0.1 and 0.2 mg/mL; Dex0.1 and Dex0.2, respectively), and DMSO were used as positive, and negative controls, respectively. Data are presented at the means ± SD. Experiments were independently done in triplicate. The symbol **** indicates significant difference (*p* < 0.0001).

All six extracts reduced plaque formation of HSV-1_WT and HSV-1_dxpiii in a dose-dependent manner at the pre-entry step. Interestingly, at 0.5 mg/mL, all six extracts significantly inhibited HSV-1_dxpiii, and reduced plaque formation in both viral strains (Figure 3 and Figure S3). In comparison, dextran (Dex) at concentrations 0.1 and 0.2 mg/mL showed 100% inhibition of plaque formation in both viral strains. These results suggest that all six extracts may interfere with the early stages of viral infection.

#### 2.4.2. Post-Entry Step

**Figure 4:**
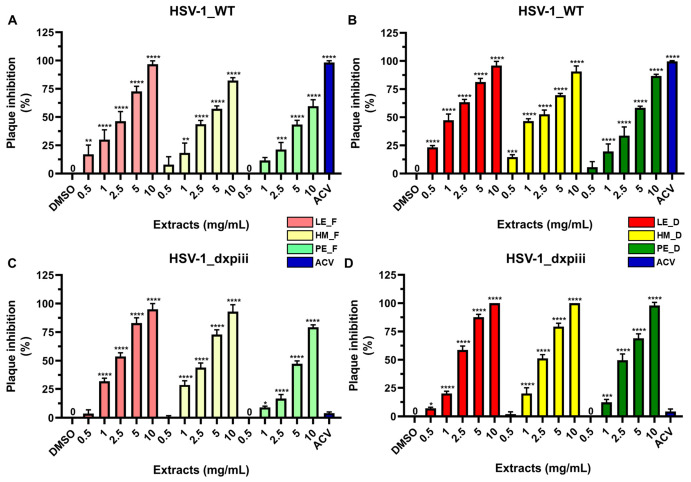
Antiviral effect of LE_F, LE_D, HM_F, HM_D, PE_F and PE_D extracts on plaque reduction in HSV-1_WT and HSV-1_dxpiii infections at the post-entry step. (**A**,**C**) Fresh and (**B**,**D**) dried extracts were treated in (**A**,**B**) HSV-1_WT and (**C**,**D**) HSV-1_dxpiii-infected cells at the post-entry step. ACV at 1 mg/mL and DMSO were used as controls. Data are presented at the means ± SD. Experiments were independently done in triplicate. The symbols *, **, ***, and **** indicate significant difference (*p* < 0.05, 0.01, 0.001, and 0.0001, respectively).

All six extracts significantly reduced viral plaque formation of HSV-1_WT and HSV-1_dxpiii in a dose-dependent manner in post-entry step (Figure 4 and Figure S4). LE_F, HM_F and PE_F had the inhibitory concentration at 50% (IC_50_), 6.06 ± 0.96, 3.76 ± 1.72, and 7.45 ± 0.50 mg/mL, respectively (Table 4). In addition, LE_D, HM_D and PE_D had IC_50_ values of 1.98 ± 0.72, 1.61 ± 1.06 and 9.55 ± 4.56 mg/mL, respectively. Among the six extracts, HM_D showed the highest selective index (SI) inhibiting HSV-1_WT, followed by LE_D and HM_F, although their SI values were substantially lower than that of ACV (743.5) (Table 4 and Figure S5). Notably, LE_F and LE_D extracts demonstrated higher SI values against drug-resistant HSV-1_dxpiii strain compared with ACV (10.66) (Table 4 and Figure S5). In particular, LE_F showed the highest SI (18.17), followed by LE_D (13.72), highlighting their potential relevance in antiviral resistance. Overall, all the six extracts demonstrate considerable antiviral activity against drug-resistant HSV-1, supporting their potential use as natural bioactive agents. Importantly, their high selectivity against the resistant strain suggests their potential as candidates for further antiviral development. Future studies should evaluate these extracts in clinically relevant models.

### 2.5. Effect of the Extracts on Viral Gene Expression and Host-Related Gene Modulation

**Figure 5:**
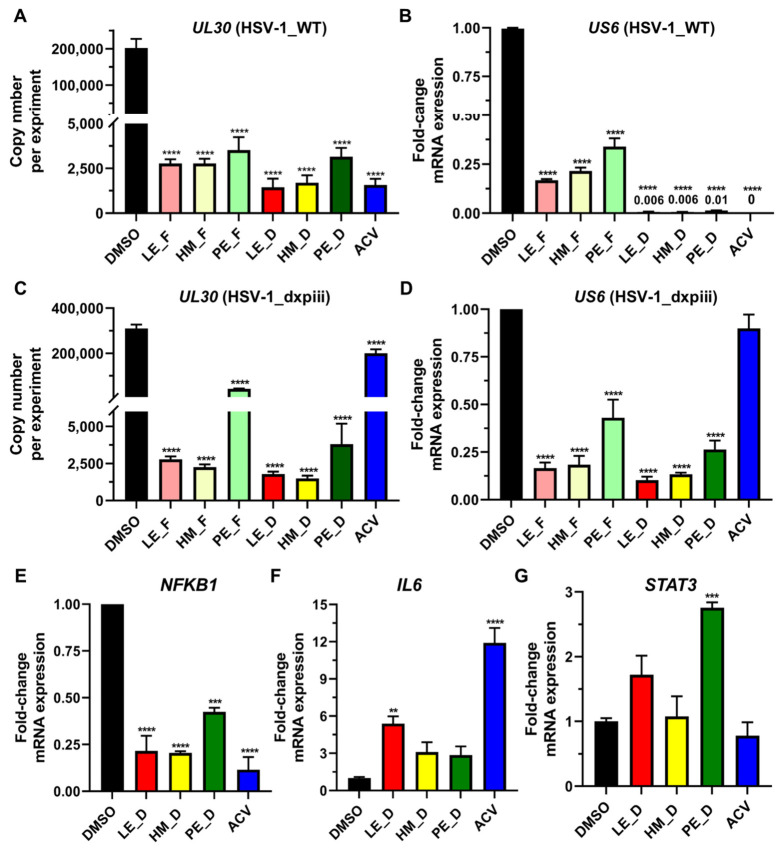
Effect of mushroom extracts on HSV-1 copy number, and viral and cellular mRNA expression in Vero cells. (**A**,**C**) *UL30* and (**B**,**D**) *US6* were detected for quantifying viral copy number and mRNA expression in (**A**,**B**) HSV-1_WT and (**C**,**D**) HSV-1_dxpiii infections, respectively. (**E**–**G**) Cellular genes *NFKB1*, *IL6*, and *STAT3* were measured in HSV-1_WT infection treated with dried extracts. ACV at 1 mg/mL and DMSO were used for the positive and negative controls, respectively. Experiments were independently done in triplicate. Data are shown as the means ± SD. The symbols **, ***, and **** indicate significant difference (*p* < 0.01, 0.001, 0.0001, respectively).

LE_D, HM_D, and PE_D reduced viral copy number (*UL30*) and HSV-1 *US6* mRNA stronger than fresh extracts (Figure 5A–D). Notably, LE_D showed the greatest suppression of both *UL30* (except in HSV-1_dxpiii) and *US6* expression, indicating that the extracts contained the potential to suppress the viral gene expression. Furthermore, the expression of host genes known to be manipulated by HSV-1 during its replication cycle was assessed. All dried extracts significantly downregulated *NFKB1*, while *IL6* expression was significantly upregulated only in LE_D, consistent with the results observed in interferon-competent infected CaSki cells (Figure 5E–G and  Figure S6). *STAT3* showed moderate induction, particularly with PE_D. While consistent trends were observed across both infected cell lines, the expression patterns of *IL6* and *STAT3* tended to differ between uninfected and infected conditions in both CaSki and Vero cells (Figures S6 and S7). Collectively, these results suggest that the mushroom extracts not only suppressed viral gene expression, but also modulated host cellular pathways including *NFKB1*, *STAT3*, and *IL6*, which are typically altered during at viral pathogenesis.

Crucially, this study provides the first evidence that LE, HM, and PE extracts exhibit potent activity against a phosphonoacetic acid- and phosphonoformate-resistant HSV-1 strain (HSV-1_dxpiii) that is also resistant to ACV [36]. All six extracts significantly reduced plaque formation of this strain at both the pre- and post-entry stages in a dose-dependent manner, suggesting that these mushrooms contain compounds that may directly target viral particles and may also act on viral DNA polymerase and host cellular proteins. High-temperature/high-pressure water extraction successfully concentrated key bioactive macromolecules, including polysaccharides, β-glucans, phenolics, and terpenoids. This enrichment, particularly in the dried extracts, was directly correlated with enhanced antioxidant activity and potent antiviral effects against both HSV-1_WT and HSV-1_dxpiii (Table 4, Figure 3 and Figure 4) [31]. A previous study demonstrated that water (room temperature) and methanol-based LE extracts derived from frozen, freeze-dried mushroom powder exhibited SI values of 14.21 and 8.2, respectively, against HSV-1 KOS, which are consistent with our observations (SI = 4.80–14.83), despite differences in extraction methods. Moreover, their extracts showed markedly higher cytotoxicity toward Vero cells (CC_50_ = 384.15 ± 12.26 to 791.97 ± 21.26 μg/mL) compared with our LE_F (CC_50_ = 29.07 ± 0.23 mg/mL) and LE_D (CC_50_ = 29.37 ± 0.39 mg/mL) (Table 4) [23]. In contrast, a dried mycelial PE extract prepared using a 0.9% NaCl solution with ultrasonic extraction showed very low inhibitory activity against HSV-2 in RK-13 cells and had a maximum tolerated concentration of only 1.55 mg/mL [37]. Collectively, our findings suggest that high-temperature/high-pressure water-based extraction may help preserve and potentially enhance the biological activity of mushroom extracts against HSV infection. To explore the mechanism in transcribed level, we examined host cell gene expression. Nuclear factor ҡB (NF-ҡB), encoded by *NFKB1,* is a central regulator of *TNF, IL1B, IL6, IL8*, as well as genes involved in cell survival and apoptosis [38]. Many viruses including HSV-1 have evolved to enhance their own replication to “hijack” [39]. Our results showed that the dried mushroom extracts significantly reduced *NFKB1* expression in both infected and uninfected cells (Figure 5, Figures S6 and S7). This finding suggests that the extracts may modulate host cellular pathways, potentially through immunomodulatory effects, which may contribute to antiviral activity against HSV-1.

### 2.6. Molecular Docking Analysis of Candidate Compounds Against HSV-1 DNA Polymerase

**Figure 6:**
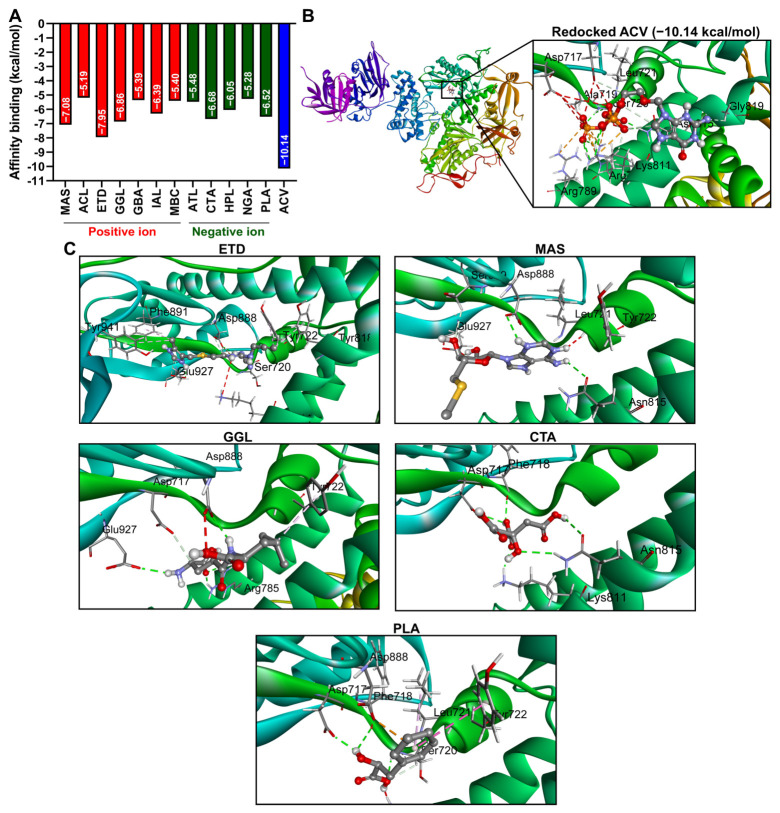
Molecular docking of top-5 predicted-candidate compounds with HSV-1 DNA polymerase. (**A**) Histogram shows the calculated affinity binding energies (ΔG, kcal/mol) of all candidate ligands in positive-ion (red bars) and negative-ion (green bars) modes. ACV (blue bar) acts as a positive control. (**B**) The binding pocket and key interactions are shown in the structure of HSV-1 DNA polymerase and its co-crystallized inhibitor ACV. (**C**) Three-dimensional interaction diagrams illustrate the docked complexes between HSV-1 DNA polymerase and candidate ligands. The grid center was fixed in the active site (x = 147.2865, y = 145.1162, and z = 123.7358) to analyze the interaction between ligand and receptor.

**Figure 7:**
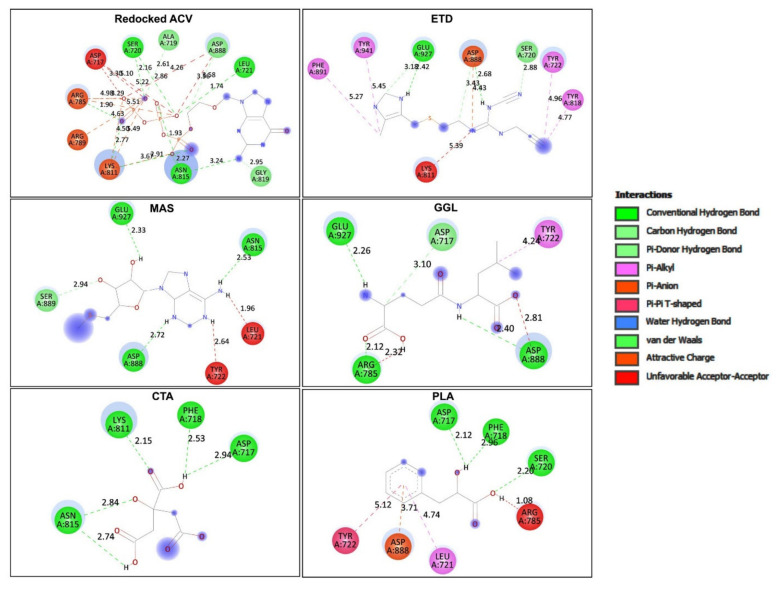
Two-dimensional interaction diagrams of the docked complexes between HSV-1 DNA polymerase and candidate ligands, ACV, ETD, MAS, GGL, CTA, and PLA. Different-colored dashed lines represent types of ligand-protein interactions, including conventional hydrogen bonds, carbon hydrogen bonds, π-donor (Pi-donor) hydrogen bonds, π-alkyl (Pi-alkyl) interactions, π-anion (Pi-anion) interactions, π-π (Pi-Pi) T-shaped interactions, water hydrogen bonds, van der Waals interactions, attractive charge interactions, and unfavorable acceptor-acceptor interactions.

Firstly, shared 26 putative compounds (Table 2 and Table 3) were initially screened against HSV-1 DNA polymerase using AutoDock Vina, applying a cut-off criterion of binding affinity >−5.0 kcal/mol (Tables S1 and S2). As a computational protocol validation, the reference standard ACV, was redocked into the active site, yielding a ΔG of −10.14 kcal/mol (Figure 6A,B). ETD (7.95 kcal/mol) showed a stronger predicted binding affinity among candidate compounds followed by 5′-methylthioadenosine (MAS) (−7.08 kcal/mol), GGL (−6.86 kcal/mol), citric acid (CTA) (−6.68 kcal/mol) and PLA (−6.52 kcal/mol) (Figure 6 and Figure 7). Simultaneously, IAL, hydroxyphenyllactic acid (HPL), acetylleucine (ATL), MBC, 4-guanidinobutyric acid (GBA), *N*-acetylglutamic acid (NGA) and acetyl-L-carnitine (ACL) show the binding energy ranked from −6.39 to −5.19 kcal/mol (Figure S8). These results demonstrated that the candidate compounds identified in the six extracts, particularly ETD, showed more promising targeting activity against HSV-1 DNA polymerase.

### 2.7. Predicted Drug-Likeness and Toxicology Properties of Candidate Compounds

Based on docking results, the 12 candidate ligands were computationally assessed for physicochemical and pharmacokinetic properties (Table 5). All compounds were predicted to satisfy Lipinski’s criteria, showing molecular weights below 500 g/mol, balanced lipophilicity (LogP < 5), and good predicted bioavailability scores (>0.17). Toxicological assessment via ProTox-3 predicted that most compounds belonged to safety Classes 4–6, signifying low toxicity. Notably, GBA, NGA, and ATL were predicted to be non-toxic (Class 6), while IAL and GGL (Class 5) had predictions comparable to ACV. Only MBC and CTA were predicted to have moderate toxicity (Class 3). These predictions suggest that the identified mushroom-derived compounds possess favorable pharmacokinetic characteristics, with ATL, GBA, GGL, IAL, and NGA being promising candidates for further experimental investigation.

Interestingly, ETD (−7.95 kcal/mol), the second most abundant compound in LE showed the strongest predicted binding affinity to HSV-1 DNA polymerase, a known target of the standard antiviral drug ACV (−10.14 kcal/mol). It interacted with the active site residues TYR722, LYS811, ASP888, and GLU927 (Figure 6 and Figure 7) [40]. MAS (−7.08 kcal/mol) bound to ASN815, ASP888, and GLU927 via conventional hydrogen bonds. GGL (−6.86 kcal/mol) interacted with ARG785, ASP888, and GLU927 through conventional hydrogen bonds. These amino acid residues play a role in directly interacting with viral DNA, indicating that these three candidate compounds identified in the six extracts may target viral DNA polymerase. Therefore, these candidate compounds possess potential antiviral activity against HSV, particularly drug-resistant strains. In addition, the twelve candidate ligands exhibited favorable drug-likeness properties based on their physicochemical and pharmacokinetic profiles (Table 5).

### 2.8. Predicted Interactions Between Metabolites and Host Signaling Pathways

**Figure 8:**
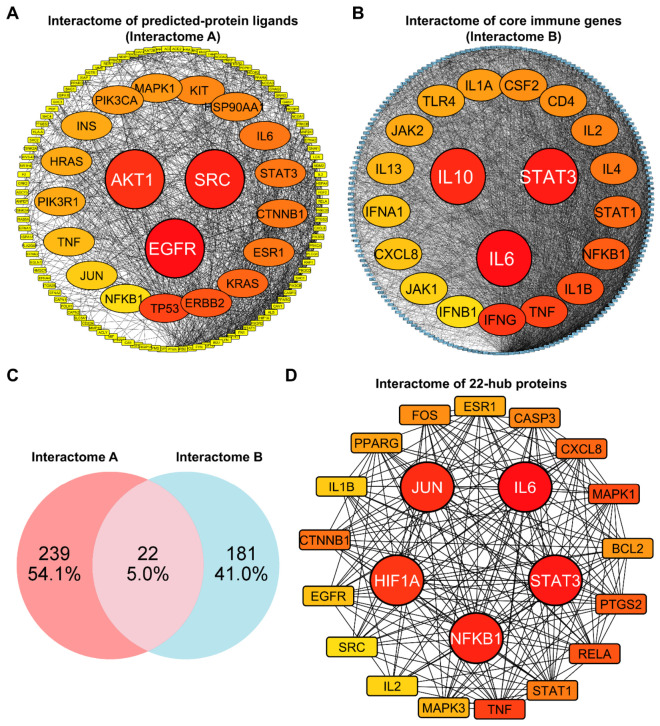
Integrated interactome and PPI analysis of host cell targets associated with the 12 candidate compounds. (**A**) Interactome A illustrates the predicted protein–ligand interaction network of the 12 candidate compounds and their associated protein targets. (**B**) Interactome B represents the core host-gene PPI network derived from key host response genes. (**C**) A Venn diagram illustrates the 22 overlapping molecular targets shared between metabolite-associated proteins in Interactome A and host-related proteins in Interactome B, indicating potential points of molecular cross-talk. (**D**) Key regulators involved in antiviral and cellular signaling pathways among the 22 hub proteins are identified using the MCC method. The node colors represent a gradient of connectivity/degree within the network, with red indicating higher centrality.

To predict the putative protein targets and biological functions of the 12 candidate ligands, an integrated ligand–target–pathway network was constructed to visualize the complex relationships among the candidate compounds, their predicted protein targets, and the associated Kyoto Encyclopedia of Genes and Genomes (KEGG) pathways (Figure S9). This network highlights how compound–protein interactions may be linked to key biological processes, suggesting potential interference with essential cellular signaling and survival pathways that are frequently exploited during viral infection. To connect the in silico target prediction with the in vitro host cell responses, we systematically integrated the predicted protein targets of the 12 candidate ligands with host-gene expression-related protein–protein interaction (PPI) networks. The predicted compound–protein targets were first combined with a STRING-derived PPI network constructed from the core host response genes, NFKB1, IL6, and STAT3. The resulting protein–ligand interactome (Interactome A; Figure 8A) comprised 261 nodes, revealing AKT1, SRC, and EGFR as highly connected hub proteins, suggesting their potential roles as major molecular mediators of ligand-associated effects. In parallel, the host-gene interactome (Interactome B; Figure 8B) consisted of 203 nodes, with IL6, IL10, and STAT3 emerging as central regulators within the host immune-related signaling network. Comparative analysis of both interactomes identified 22 overlapping protein targets (Figure 8C), indicating molecular convergence between metabolite-associated targets and host response pathways. Subsequent network centrality analysis using the Maximal Clique Centrality (MCC) method highlighted STAT3, NFKB1, JUN, HIF1A, and IL6 as the most influential hub proteins within the integrated interactome (Figure 8D). These proteins are known to play critical roles in antiviral signaling, inflammatory responses, and cellular stress regulation [41,42,43,44]. These findings demonstrate that metabolites derived from LE, HM, and PE may exert antiviral activity through a multi-target network-based mechanism, modulating key host cell signaling pathways that are commonly exploited during HSV-1 infection.

### 2.9. Core Hub Proteins Involved in Host Cell Responses

**Figure 9:**
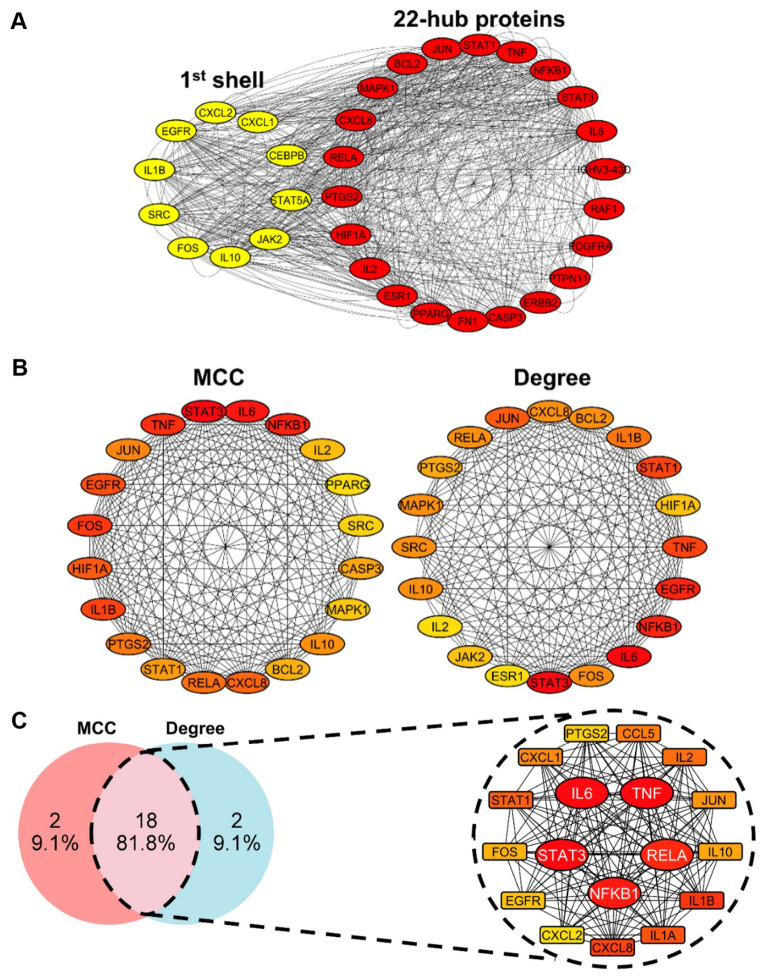
Identification of core hub proteins involved in host cell responses. (**A**) The PPI network constructed from 22 hub proteins (red) and their 1st shell interactors (yellow). (**B**) Core sub-networks were derived from the initial PPI network using MCC algorithm, which identifies essential nodes within highly interconnected clusters, and Degree centrality, which ranks nodes based on the number of direct connections (edges), implemented in cytoHubba plugin. The node color gradient from red to yellow represents the ranking from highest to lowest scores, respectively. (**C**) Venn diagram illustrating the 18 hub proteins identified by the intersection of both algorithms along with the interconnected sub-network of the 18 hub proteins. The node colors (red, orange, yellow) represent the gradient of their degree/MCC scores. In the Venn diagram, the red and blue circles denote the MCC and Degree methods, respectively.

After identifying the 22 overlapping hub proteins from the intersection of Interactome A and Interactome B, an extended PPI network was constructed to further characterize the functional relationships among these targets (Figure 9A). This extended network includes the 22 hub proteins (red nodes) together with their first-shell interactors (yellow nodes), allowing the identification of both direct and indirect interaction patterns within host immune- and signaling-related pathways. To prioritize the most influential nodes within this extended network, topological analyses were performed using two complementary centrality algorithms, MCC and Degree, implemented in the cytoHubba plugin (Figure 9B). Both approaches consistently highlighted a subset of highly connected and topologically important proteins, indicating robust network centrality independent of the algorithm used. Intersection analysis of the MCC- and Degree-ranked proteins identified 18 core hub proteins, representing 81.8% of the initially overlapping targets (Figure 9C). These core hubs form a densely interconnected subnetwork characterized by extensive cross-talk among inflammatory and antiviral signaling components. Notably, IL1B, TNF, IL6, RELA, NFKB1, and STAT3 emerged as dominant hubs within this core network, exhibiting high connectivity and central positioning. These proteins are well recognized as key regulators of cytokine signaling, NF-κB activation, and host antiviral and inflammatory responses, suggesting that they may serve as important molecular nodes through which the candidate compounds potentially modulate host cell signaling during HSV-1 infection.

### 2.10. Gene Ontology (GO) and KEGG Enrichment and the Core Hub Protein-Associated Enriched Pathways

**Figure 10:**
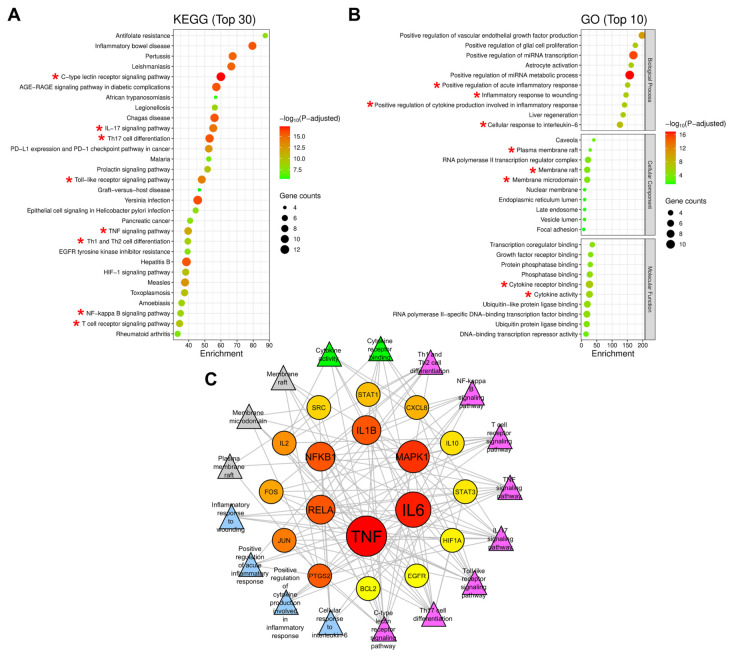
Go and KEGG enrichment analysis of key targets and the core hub protein-associated enriched pathways. (**A**) Top 30 enriched KEGG pathways and (**B**) top 10 enriched GO including BP, CC and MF terms illustrate the possible mechanism of candidate compounds against HSV-1. (**C**) A network visualizing the relationships between the core hub proteins and their associated enriched pathways. The purple, blue, green and gray triangles represent KEGG, BP, CC, and MF, respectively. Asterisks (*) indicate genes/proteins/pathways associated with immune-related functions.

KEGG enrichment analysis of these 18 core proteins revealed significant involvement in key cellular signaling pathways, including IL17, TNF, and NF-κB signaling pathways (Figure 10A), which are associated with inflammatory and cellular response and are often implicated in viral infections. Consistent GO with biological process (BP), cellular component (CC) and molecular function (MF) enrichment results indicated roles in cytokine activity, transcriptional regulation, and cellular responses (Figure 10B). Taken together, TNF, IL6, MAPK1, IL1B, NFKB1, RELA, and STAT3 represent key mediators and potential therapeutic targets for interfering with virus–host interactions (Figure 10C). Integrated network and enrichment analyses suggest that these proteins form a coordinated regulatory system. Based on our experimental findings showing that the extracts modulated *NFKB1*, *IL6*, and *STAT3* expression in uninfected Vero and CaSki cells, along with in silico analysis, the mushroom’s candidate metabolites may exert immunomodulatory effects on host cellular pathways.

### 2.11. Predicted Interaction of Candidate Targets and Mushroom-Derived Compounds with Hub Proteins

**Figure 11:**
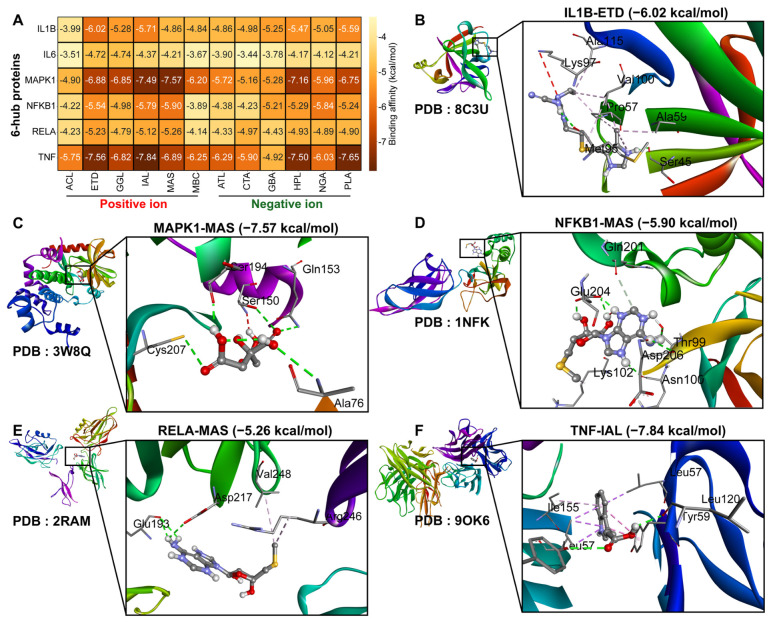
Molecular docking analysis of six hub proteins. (**A**) A heatmap illustrates the binding affinities of the 12 candidate ligands against the hub proteins. The molecular docking poses of (**B**) IAL with TNF, (**C**) MAS with MAPK1, (**D**) ETD with IL1B, (**E**) MAS with NFKB1, and (**F**) MAS with RELA are shown as 3D interaction representations. The grid centers were fixed in the active site of IL1B (x = 4.02, y = −13.13, and z = −20.3), IL6(x = 7.28, y = 20.19, and z = 7.3), MAPK1 (x = 29.29, y = 10.71, and z = 13.7), NFKB1 (x = −11.05, y = 7.41, and z = 18.83), RELA (x = 11.04, y = 26.48, and z = 52.40) and TNF (x = 51.90, y = −17.57, and z = 36.1).

**Figure 12:**
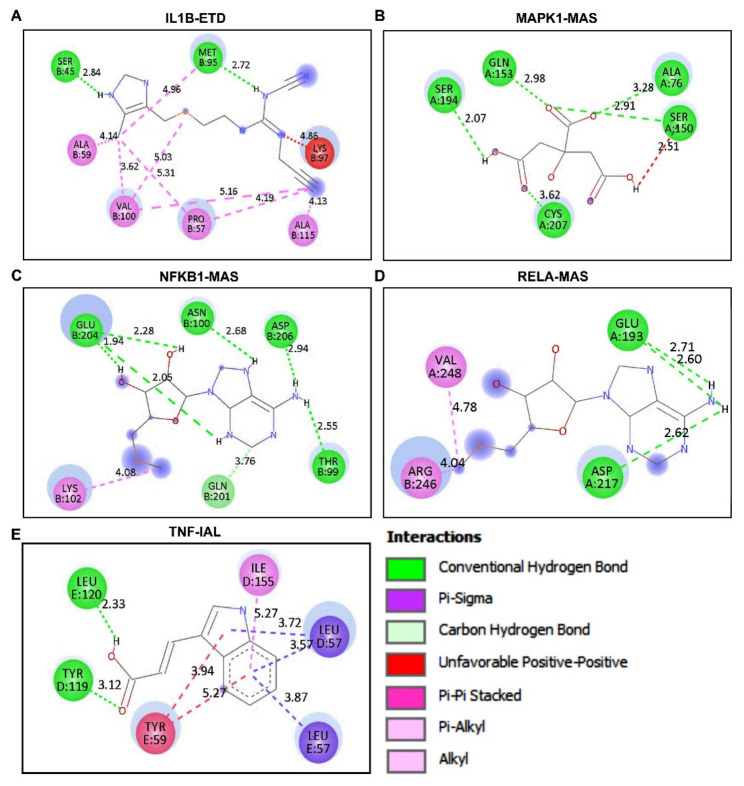
Two-dimensional interaction of the molecular docking poses in six hub proteins and ligands. The molecular docking poses of (**A**) ETD with IL1B, (**B**) MAS with MAPK1, (**C**) MAS with NFKB1, (**D**) MAS with RELA, and (**E**) IAL with TNF are shown as 2D structures. Different-colored dashed lines represent types of ligand-protein interactions, including conventional hydrogen bonds, carbon-hydrogen bonds, unfavorable acceptor-acceptor interactions, π-sigma (Pi-sigma) interactions, π-alkyl (Pi-alkyl) interactions, π-π (Pi-Pi) stacked interactions, and alkyl interactions.

To further investigate the interactions between the 12 candidate ligands and the six key hub proteins, molecular docking was performed (Figure 11). The heatmap of predicted binding affinities showed that IAL, MAS, and ETD exhibited interactions with several targets, including TNF, MAPK1, NFKN1 and RELA, and IL1B (Figure 11 and Figure 12). MAS demonstrated potential multi-target affinities with MAPK1 (−7.57 kcal/mol), NFKB1 (−5.90 kcal/mol), and RELA (−5.26 kcal/mol). ETD showed predicted binding to IL1B (−6.02 kcal/mol) and TNF (−7.56 kcal/mol), while IAL, PLA, and HPL displayed affinities toward TNF and MAPK1 (Figure 11A). MAS was predicted to interact with MAPK1 and RELA with hydrogen bounds, while ETD exhibited potential binding to IL1B through multiple non-covalent interactions (Figure 11B–F and Figure 12A–E). Overall, these docking results support the multi-target binding potential of these candidate metabolites, and provide a computational basis for their possible interactions with host-related proteins. However, further experimental validation using purified compounds is required to conform their biological activities.

Based on GO/KEGG enrichment of core hub proteins, TNF, IL6, IL1B, NFKB1, RELA, and STAT3 were identified as key molecular targets associated with the mushroom extracts and potential therapeutic targets for interfering with virus–host interactions (Figure 9). Correspondingly, Abou-Taleb et al. [25] identified MAPK1, SRC, EGFR, JAK1, IKBKB, AKT1, TNF, MMP9, and IL6 as centrally nodes in PPI map, supporting their potential involvement in HSV pathogenesis. Liu et al. [26] also elucidated 36 hub proteins associated with our finding (e.g., MAPK1, STAT3, IL1B, and IL6). The in silico analysis suggests that these mushroom-derived candidate metabolites may contribute to antiviral activity by targeting not only viral proteins but also multiple host factors. For example, predicted compounds like MAS, ETD, and IAL were associated with host proteins, including NFKB1, TNF and MAPK1, which are involved in key cellular signaling pathways [45]. Mechanistically, MAPK1 and NFKB1, key regulators of inflammatory and antiviral pathways, are activated by HSV during early infection. This activation contributes to viral evasion of host immune responses and promotes viral survival, as well as facilitating viral gene expression and replication [46]. These pathways are also known to regulate downstream IL6 and STAT3, which play roles in both inflammatory responses and antiviral defense [46]. In addition, inactivation of IL6 and STAT3 facilitates HSV infection and increases the symptom severity [36,47]. Predicted metabolite binding to MAPK1 may influence its phosphorylation status or downstream signaling, which may modulate transcription factors for IL6 and STAT3 expression. Similarly, predicted interactions with NFKB1 may alter its stability or PPI, which could in turn affect downstream signaling pathways and gene expression in infected cells. However, these interpretations remain speculative, as the in silico findings reflect binding potential rather than direct functional or transcriptional outcome. Notably, the consistency between the predicted targeting of these candidate proteins and the observed changes in vitro assay supports a possible link between compound activity and host signaling modulation, as well as direct inhibition of HSV, thereby contributing to antiviral activity. Furthermore, the integration of molecular docking with network pharmacology enhances the biological relevance of the computational predictions. Together, cross-validation between the in silico network and the in vitro gene expression data supports the hypothesis that the mushroom-derived metabolites may exert antiviral effects through coordinated multi-target interactions involving both viral and host cellular mechanisms. Major enriched pathways, including NF-κB, TNF, and IL17 signaling, are known to be exploited by HSV-1 for replication, suggesting that these metabolites may interfere with host hijacking mechanisms [39,48].

This study has certain limitations. First, the crude extracts exhibited modest antiviral activity against HSV-1_WT, with lower potency than ACV, which is typical of complex compounds and may be attributed to inefficient intracellular delivery. However, the LE_F and LE_D extracts showed a trend toward higher inhibitory activity against HSV-1_dxpiii than ACV (Table 4). Therefore, further development should focus on formulation strategies, such as liposomes, polymeric nanoparticles, or lipid-based delivery systems, to overcome intracellular delivery barriers and enhance cellular uptake and antiviral efficacy. In addition, synergistic combinations of the extracts with ACV should be further investigated against drug-resistant strains. Second, a gap exists between in silico predictions and in vitro validation, as the biological assays were conducted using crude extracts. Consequently, the roles of the predicted compounds (MAS, ETD, and GGL) remain hypothetical and require further validation through purified-compound in vitro and in vivo studies. Additionally, to further validate these predicted interactions, studies may employ biophysical approaches such as Surface Plasmon Resonance or Isothermal Titration Calorimetry to determine binding affinities. In addition, Western blot analysis could be used to assess downstream signaling effects, including changes in protein phosphorylation (e.g., MAPK1 activation) and transcription factor nuclear translocation. Third, although the high-temperature and high-pressure (autoclave) extraction method effectively disrupts the rigid cell walls of the mushroom and may increase the yield of water-soluble polysaccharides, such extreme conditions may also promote the thermal degradation of certain heat-sensitive, low-molecular-weight bioactive compounds, including phenolics, flavonoids, and terpenoids [49,50,51]. The antiviral activity observed in this study may be attributable to heat-stable compounds. In contrast, milder extraction methods, such as aqueous or organic solvent extraction at room temperature, as well as ultrasound-assisted, or enzyme-assisted techniques could be further explored as alternative strategies to better preserve those metabolites which may contribute differently to anti-HSV activity compared with the present extracts [50,52]. However, milder extraction methods may also retain heat-labile toxins or cytotoxicity-associated compounds, which could elevate the cytotoxicity relative to extracts obtained under extreme conditions. Finally, it should be noted that metabolite annotation in this study was based on LC-ESI-QTOF-MS/MS data and spectral matching against databases, which represents putative identification rather than definitive compound confirmation. Further confirmation of compound identification and quantification would require additional analytical approaches, such as comparison with authentic reference standards using targeted liquid chromatography-tandem mass spectrometry (LC-MS/MS) analysis, as well as complementary techniques including NMR spectroscopy. Moreover, this study lacks an unprocessed (fresh/raw) mushroom control group, which restricts a direct comparison of how high-temperature autoclaving and dehydration affect the native metabolomics profile. Incorporating raw mushroom controls in future studies will be important to better understand the chemical changes induced by these processing methods. Despite these limitations, the comparative differences in metabolite profiles across six extracts remain meaningful for comparative analysis. Overall, the present in vitro and in silico findings do not fully represent the complete antiviral potential of the six commercial mushroom extracts against HSV. Moreover, differences in extraction methods and mushroom sources may substantially influence the observed biological activities and metabolite profiles of these mushrooms. 

## 3. Materials and Methods

### 3.1. Preparation of Mushroom Extracts

Fresh LE, HM, and PE were purchased from a supermarket in Bangkhen District, Bangkok, Thailand (13°52′46.4′′ N 100°36′00.3′′ E), during November 2024. These mushroom cultivars are officially certified by the Ministry of Agriculture and Cooperatives, Thailand (Figure S10). For fresh extracts (LE_F, HM_F, and PE_F), 200 g of each species was autoclaved with Milli-Q water at 121 °C for 1 h to enhance extraction of bioactive compounds. For dried extracts (LE_D, HM_D, and PE_D), 200 g of mushrooms were baked at 65 °C for 4 days, then extracted under the same conditions. All extracts were lyophilized to yield dry powders, which were subsequently dissolved in DMSO to prepare a 1000 mg/mL stock solution. At this concentration, the stock solutions exhibited a highly viscous, paste-like consistency, but were fully homogenized via intense vortexing and warming at 50 °C for 15 min. To ensure sterility and remove any remaining particulates, the solutions were filtered through a 0.2 μm polytetrafluoroethylene membrane filter (Polydisc TF, Whatman, Inc., Clifton, New Jersey, USA) before being diluted in the culture medium for subsequent bioassays.

### 3.2. Bioactive Compound and Antioxidant Activities

#### 3.2.1. Total Polysaccharides by Phenol-Sulfuric Acid Method

The extract at 60 µL was mixed with 60 µL of 5% phenol. The mixture was added with 300 µL of H_2_SO_4_, then mixed thoroughly, and subsequently was aliquoted into a 96-well plate (100 µL/well) in triplicate. The polysaccharide was measured using colorimetric analysis at 540 nm (Multiskan^TM^ microplate photometer; Thermo Fisher Scientific, Waltham, MA, USA), according to [53] and compared with a standard curve of glucose.

#### 3.2.2. α- and β-Glucan Contents by Calorimetric Method

The β-(1,3-1,6)-D-glucan content was determined using a β-glucan assay kit K-YBGL (yeast and mushrooms) from Megazyme International Ireland Ltd., (Bray, Ireland), according to the manufacturer’s protocol. Briefly, each of the powder extracts (500 mg) was solubilized and hydrolyzed using ice-cold 12 M H_2_SO_4_ for 2 h and then boiled at 100 °C for 2 h. The solutions were neutralized with 8 M NaOH and 200 mM NaOAc pH 4.5. The total glucan contents were measured by adding 0.1 mL of the mixture with exo-1,3-β-glucanase mixed with β-glucosidase, followed by incubation at 40 °C for 1 h. The glucose oxidase-peroxidase (GOPOD) reagent was added to the mixture and continuously incubated at 40 °C for 20 min. Then, the absorbance was measured at 510 nm. The α-glucan content was measured by adding each extract with 1.7 M NaOH on ice for 20 min, followed by amyloglucosidase mixed with invertase and trehalase, which was incubated at 40 °C for 1 h. Then, 0.1 mL of each solution was added with GOPOD and incubated at 40 °C for 20 min. Next, the absorbance was measured at 510 nm. D-glucose was used for the standard solution. The β-glucan content was calculated using the formula: β-Glucan content = [(Total glucan content) − (α-Glucan content)] [54].

#### 3.2.3. Total Phenolic Compounds by Vanillin Acid Method

Total phenolic compounds (TPC) assay was performed according to a previous study [55], with modification. Briefly, each extract (100 µL) was mixed with 300 µL of the solution containing 0.1 mg/mL vanillin in 70% sulfuric acid (*v*/*v*). Then, the mixtures were incubated at room temperature for 20 min in the dark and subsequently aliquots (100 µL/well) were placed in a 96-well plate in triplicate. The absorbance was measured at a wavelength of 500 nm. Tannin (0–100 mg/mL) was used to prepare a standard curve.

#### 3.2.4. Total Terpenoids Assay

Each extract (500 µL) was mixed with 3.5 mL of ice-cold 95% methanol. Then, 400 µL of the extract was added to 750 µL of chloroform and incubated for 3 min. H_2_SO_4_ (200 µL) was loaded into the mixture and incubated at 25 °C for 1.5 h in the dark. A 200 μL reddish-brown layer was collected after removal of the upper layer and subsequently mixed with 750 μL of 95% methanol. The mixture (100 μL/well) was loaded into a 96-well plate and measured at 538 nm. Concentrations (0–1.191 mg/mL) were calculated using a standard curve of linalool (Alfa Aesar; Ward Hill, MA, USA).

#### 3.2.5. Total Crude Protein

Each extract (20 µL) was incubated with 200 µL of 1× Bradford protein assay (Bio-Rad, Hercules, CA, USA) and measured at 490 nm. The extract samples were prepared in triplicate. The concentrations were determined from bovine serum albumin at concentrations of 0, 1.25, 2.5, 5, 10, 15, 20 and 25 mg/mL as the standard curve.

#### 3.2.6. 2,2-Diphenyl-1-Picrylhydrazyl (DPPH) Radical Scavenging Assay

DPPH radical scavenging assay of the extracts was determined by modifying a previous study [56]. One milliliter of extract (50 mg/mL) was mixed with 1 mL of 0.1 mM DPPH solution (SigmaAldrich, St. Louis, MO, USA). The mixture was kept in the dark at room temperature for 30 min, and the absorbance at 517 nm was measured with a spectrophotometer (Genesys 180, Thermo Fisher Scientific, Waltham, MA, USA). The DPPH radical scavenging activity of the extract was expressed in micrograms of gallic acid equivalent (GAE) per gram of dried extract.

#### 3.2.7. Ferric Reducing Antioxidant Power (FRAP) Assay

FRAP assay was performed as previously described [57]. Briefly, 100 µL of the extract (50 mg/mL) was mixed with 3 mL of FRAP reagent. Then, the reaction mixture was incubated at 37 °C for 4 min. Subsequently, the absorbance was determined at 593 nm with a spectrophotometer (Genesys 180, Thermo Fisher Scientific). The FRAP reagent (SigmaAldrich, St. Louis, MO, USA) was prepared from 300 mM sodium acetate buffer (pH 3.6) (Merck, Darmstadt, Germany), 10 mM 2,4,6-Tris(2-pyridyl)-s-triazine (TPTZ) solution (Merck, Darmstadt, Germany) and 20 mM ferric chloride solution (Merck-Schuchardt, Hohenbrunn, Germany) in a ratio of 10:1:1 (*v*/*v*), respectively. The FRAP reagent was freshly prepared and warmed to 37 °C before use. Results were expressed as micrograms of GAE per gram of dried extract.

### 3.3. Determination of Metabolomic Profiles

Untargeted metabolite profiling of the extracts was performed using the ESI-QTOF-MS. The analysis was conducted at the Proteomics Services, Faculty of Medical Technology, Mahidol University (Bangkok, Thailand). Briefly, the six extract powders were dissolved in 0.1% formic acid and filtered through 0.45 µm hydrophilic nylon syringe filter. A 3 µL aliquot was injected into a DIONEX Ultimate 3000 HPLC (Dionex Softron, GmbH, Germany) equipped with an Acclaim Advantage II C18 column (2.1 × 100 mm, 3 μm) and guard column (3 × 10 mm, 5 μm). The column and autosampler temperatures were set at 40 °C and 10 °C, respectively. Metabolites were detected using a Bruker compact QTOF-MS (Bruker, Germany) in positive-ion and negative-ion modes (*m*/*z* 50–1000) with nebulizer pressure of 2 bars, drying gas flow 8 L/min, and temperature 220 °C. The mobile phase to separate the chromatographs consisted of water with 0.1% forming acid (A) and acetonitrile with 0.1% forming acid (B), delivered at a flow rate 0.3 mL/min under gradient elution: 99% A at 0–2 min, gradually shifting to 99% B by 17–20 min, followed by 99% B back to 99% A between 20 and 20.1 min. From 20.1 to 28.5 min, the mobile phase was maintained at 99% A and 1% B with a flow rate of 0.35 mL/min, followed by a final step from 28.5 to 30 min at the same composition with a flow rate of 0.25 mL/min. Sodium formate served as the external calibration standard. Acquisition was performed in two segments, including an initial auto MS scan (0–0.3 min) for sodium formate calibration, followed by auto MS/MS fragmentation (0.3–30 min). Data acquisition was conducted in both positive and negative ionization modes with a data acquisition rate of 12 Hz. The mass scan range was set from *m*/*z* 20–1300. The precursor ion intensity threshold was adjusted to 0.5, with the number of precursor up to three and the cycle time to 0.5 s. An intensity threshold of 400 counts was applied for precursor selection. Active exclusion was applied for three spectra and released after 0.2 min. Data were analyzed using MetaboScape^®^ 2022 (Bruker Daltonics GmbH & Co. KG, Bremen, Germany). Briefly, raw data of LC-MS/MS were processed using MetaboScape^®^ 2022 (Bruker Daltonics) with the T-ReX 3D workflow. The feature extraction parameters were set as follows: intensity threshold of 1000 and minimum peak length of 7 spectra, with peak area used for feature quantification. Mass calibration was performed within the 0–0.3 min retention time window. The auto MS/MS scan was processed using the average method, covering a retention time range of 0.3–25 min and a mass range of 50–1000 m/z. Metabolite annotation was performed by matching MS/MS spectra and retention times against the Bruker MetaboBase Personal Library 2.0 and the MONA database. Compounds were annotated through library matching, and metabolites with higher annotation quality scores were selected based on retention time, MS/MS score, m/z values, mSigma, and analyte spectral library matching. Quantification was based on metabolite peak intensities. Only statistically significant metabolites (*p* < 0.05) with confirmed matches in the Bruker MetaboBase Personal Library 2.0 and MONA databases were included in the final dataset. The processed metabolite datasets were subsequently exported as CSV files for further analysis.

### 3.4. In Vitro Study

#### 3.4.1. Cell Culture

Vero cells (African green monkey kidney; kindly provided by Prof. Dr. Pilaipan Puthavathana, Mahidol University, Thailand), and CaSki (HPV-16-positive cervical cancer cells; kindly obtained from Prof. Dr. Tohru Kiyono, National Cancer Center Research Institute, Tokyo, Japan) were maintained in DMEM supplemented with 10% fetal bovine serum (Gibco; Thermo Fisher Scientific; Waltham, MA, USA), 2.5 µg/mL amphotericin B, 100 U/mL penicillin and 100 µg/mL streptomycin at 37 °C in 5% CO_2_ [36].

#### 3.4.2. Evaluation of Cell Viability in Response to Mushroom Extracts

Vero cells (2,500 cells/well in a 96-well plate) were incubated for 24 h, followed by 72 h treatment with serial extracts. Subsequently, 10 μL of 5 mg/mL MTT (Invitrogen; Carlsbad, CA, USA) was added to the cells and incubated for 4 h. Optical density (OD) at 540 nm was measured, and cell viability (%) calculated as: Cell viability (%) **=** [1 − (OD treatment/OD untreated)] × 100. The cytotoxic concentration at 50% (CC_50_) was determined from the dose–response curve generated by plotting % cell viability and extract concentrations using nonlinear regression analysis. CC_50_ values were expressed in mg/mL and presented as mean ± SD.

#### 3.4.3. Virus Strains and Propagation

HSV-1 strains, including KOS (a thymidine kinase-positive and the ACV-sensitive stain; HSV-1_WT) and dxpiii (phosphonoacetic acid and the phosphonoformate-resistant strain; HSV-1_dxpiii) were generously provided by Prof. Donald Coen (Biological Chemistry and Molecular Pharmacology, Harvard Medical School, Boston, USA), and propagated in Vero cells. Viral titers were determined by 10-fold serial dilution using plaque assay (Figure S11). The viral stocks were kept at −80 °C until use. All experiments involving HSV-1 were conducted in a Biosafety Level 2 (BSL-2) laboratory in accordance with institutional biosafety regulations for Risk Group 2.

#### 3.4.4. Assessment of the Extracts on the Viral Infection During Pre-Entry Step

Vero cells (100,000 cells/well) were seeded into 24-well plates and incubated for 24 h. The extracts at different sub-toxic concentrations were mixed 1:1 (*v*/*v*) with HSV-1_WT or HSV-1_dxpiii at multiple of infection (MOI) 0.002, resulting in a final MOI of 0.001 for 1 h at 37 °C. The cells were then exposed to the virus–extract mixture for 2 h. After removal of unbound virus, extracts at indicated concentrations were added in complete media with 0.5% carboxymethyl cellulose (CMC). ACV (1 mg/mL), Dex (0.1 and 0.2 mg/mL) and DMSO served as positive and negative controls, respectively. The infected cells were incubated for 72 h until the plaque appeared clearly. Plaques were fixed with 10% formaldehyde, stained with 0.5% crystal violet, and counted. The percentage of viral plaque inhibition (%) was calculated using the formula: Plaque inhibition (%) = [1 − (Number of plaques in treatment/Number of plaques in viral control DMSO)] × 100.

#### 3.4.5. Assessment of the Extracts on the Viral Infection During Post-Entry Steps

Vero cells (100,000 cells/well) were seeded into 24-well plates and incubated for 24 h. The cells were then infected with HSV-1_WT or HSV-1_dxpiii at an MOI 0.001 for 2 h. After removal of unbound virus, extracts at indicated concentrations were added in complete media with 0.5% CMC. ACV (1 mg/mL) and DMSO served as positive and negative controls, respectively. The infected cells were incubated for 72 h until plaque occurred clearly. Plaques were fixed with 10% formaldehyde, stained with 0.5% crystal violet, and counted. The percentage of viral plaque inhibition (%) was calculated using the formula: Plaque inhibition (%) = [1 − (Number of plaques in treatment/Number of plaques in viral control DMSO)] × 100. The IC_50_ value was calculated from the dose–response curve generated by plotting % cell viability against extract concentration, using nonlinear regression analysis. IC_50_ values were expressed in mg/mL and presented as mean ± SD.

#### 3.4.6. Modulation of Viral and Host-Related Gene Expression by Mushroom Extracts

RNA was extracted from HSV-1-infected and uninfected Vero and CaSki cells with mushroom extracts using TRIzol™ reagent (Thermo Scientific, Waltham, MA, USA). DNA was isolated from HSV-1-infected Vero cells using GeneAll^®^ Exgen™ and Trizol, respectively. Viral DNA (*UL30*) copy number was quantified by qPCR with SsoAdvanced™ SYBR^®^ Green on an Eco48 system, using 40 ng DNA and a standard curve from 10^1^ to 10^8^ HSV-1 copies. For gene expression, cDNA was synthesized from total RNA using RevertAid First Strand Kit, and expression of viral genes (*US6*) and host-related genes (*NFKB1*, *IL6*, *STAT3*) was determined by qRT-PCR with *GAPDH* as internal control. Relative expression was calculated using the 2^−ΔΔCT^ method. Primer sequences are listed in Table S3.

### 3.5. In Silico Study

#### 3.5.1. Metabolic Profile Analysis

Metabolic profiles including heat maps, PCA score plots, top 25 enriched metabolite bars and pie charts of the identified bioactive compounds from the six mushroom extracts derived from ESI-QTOF-MS were analyzed using Metaboanalyst 6.0 web-based software (https://www.metaboanalyst.ca/) (accessed on 10 October 2025) [58].

#### 3.5.2. Molecular Docking Assay

HSV-1 DNA polymerase (PDB 8V1T) was retrieved from the Protein Data Bank (https://www.rcsb.org/ accessed on 13 October 2025), while ligand structures of ACL, ATL, CTA, ETD, GBA, GGL, HPL, IAL, MAS, MBC, NGA, and PLA were obtained from NCBI PubChem. Proteins and ligands were prepared by removing water, adding hydrogens, assigning Gasteiger charges, and generating molecular surfaces using AutoDock Tools (Version 1.5.7) [59] and underwent screening with AutoDock Vina (Version 1.2.3) [60] with a consideration of free-binding energy < −5.0 kcal/mol and RMSD < 2 Å. The molecular docking search was centered on the active site of the HSV-1 DNA polymerase, with the grid center coordinates fixed at x = 147.2865, y = 145.1162, and z = 123.7358. The grid box dimensions were set to x= 20, y = 22, and z = 38 Å with a grid spacing of 0.375 Å with an exhaustiveness value of 32 to adequately cover the binding pocket and allow for flexible ligand orientation. Ligand–protein interactions were visualized in 2D and 3D using BIOVIA Discovery Studio (Version 25.1.0).

#### 3.5.3. Absorption, Distribution, Metabolism and Excretion Analysis and Toxicology

The candidate components, identified through molecular docking against HSV-1 DNA polymerase (Section 3.5.2), were subjected to the SwissADME database (http://www.swissadme.ch/) (accessed on 20 October 2025). Drug-likeness was assessed based on established criteria, including Lipinski’s rule of five and Veber’s rule [61,62]. Furthermore, the components were analyzed for potential toxicology using the ProTOX 3.0 database (https://tox.charite.de/protox3/)(accessed on 20 October 2025) [63].

#### 3.5.4. Network Pharmacology Analysis and Prediction of Bioactive Compounds

The candidate 12-docked ligands against HSV-1 DNA polymerase were subjected to the SwissTargetPrediction (https://www.swisstargetprediction.ch/) (accessed on 14 November 2025) and PharmMapper (https://www.lilab-ecust.cn/) (accessed on 14 November 2025) databases to predict potential off-target effects. A compound–target–host protein interaction network was conducted using STRING (https://string-db.org/) (accessed on 14 November 2025). The network parameters were set to a minimum required confidence score of >0.7 and included first shell interactors, limited to no more than 5 or 10 per node. The network was subsequently visualized and analyzed using Cytoscape (Version 3.10.4) [64] with the cytoHubba plugin for hub protein identification [65]. All obtained genes targets within the network were subjected to KEGG and GO enrichment using the R software (Version 4.4.2) with the “clusterProfiler” package [66] and the “org.Hs.eg.db” package utilized as the human universal gene set. Pathways were considered statistically significant with *p* < 0.01. The *p*-values were adjusted for multiple testing using the Benjamini–Hochberg method and fold enrichment values were calculated to indicate the degree of overrepresentation of the genes. To validate the network pharmacology predictions, site-directed molecular docking was performed between the identified candidate ligands and six predicted hub proteins, including interleukin 1B (IL-1B: PDB 8C3U), interleukin-6 (IL-6: PDB: 4NI9, nuclear factor kappa B (NFKB: PDB 1NFK), mitogen-activated protein kinase 1 (MAPK1: PDB 3W8Q), REL-associated protein involved in NF-κB heterodimer formation, nuclear translocation and activation (RELA: PDB 2RAM, tumor necrosis factor (TNF: PDB 9OK6) (https://www.rcsb.org/ accessed on 20 November 2025). Docking simulation was executed using Autodock Vina with site-specific grid boxes. The docking searches were defined by site-specific grid boxes for the six protein targets. The grid centers (x, y, z) and size dimensions (x, y, z) in Å were configured as followed: IL1B (center: 4.02, −13.13, −20.3; size 20, 20, 20), IL6 (center: 7.28, 20.19, 7.34; size 20, 20, 20), MAPK1 (center: 29.29, 10.71, 13.67; size 20, 20, 22), NFKB1 (center: −11.05, 7.41, 18.83; size 30, 24, 24), RELA (center: 11.04, 26.48, 52.40; size 34, 30, 38), and TNF (center: 51.92, −17.57, 36.1; size 20, 24, 24). All grid parameters were set with a grid spacing of 0.375 Å with an exhaustiveness value of 32 to adequately cover the binding pocket and allow for flexible ligand orientation.

### 3.6. Statistical Analysis

All data were presented as mean ± SD values. The experiments were performed in at least triplicate (*n* ≥ 3). For relative gene expression and inhibition analyses, data were normalized to the vehicle control group (DMSO). This normalization mathematically fixed the control baseline to 1.00 for gene expression and 0.00% for inhibition (%), making its variance zero by default. To accommodate this normalization constraint, statistical significances were analyzed using ordinary one-way ANOVA followed by Dunnett’s multiple comparison test to evaluate difference between each treatment group and the fixed control. Separately, to compare yields of candidate bioactive compounds, antioxidant activities, CC_50_, and IC_50_ among six extracts without a control group, statistical differences were evaluated using one-way ANOVA followed by Tukey’s multiple comparison test. Statistical analysis was performed using the Graphpad Prism software version 9.0 (San Diego, CA, USA). Differences were considered statistically significant at *p* < 0.05 and asterisks indicated *p* < 0.05 (*), *p* < 0.01 (**), *p* < 0.001 (***), and *p* < 0.0001 (****). The exact *p*-value of all comparisons is shown in Table S4.

## 4. Conclusions

In conclusion, this study provides preliminary scientific support for the traditional medicinal use of LE, HM, and PE by demonstrating that their high-temperature/high-pressure water extracts, particularly from dried samples, are rich in bioactive metabolites and exhibit significant antiviral activity against both wild-type and drug-resistant HSV-1 strains in vitro. Integrated in vitro and in silico analyses suggest the potential for a multifaceted antiviral mechanism that may involve direct inhibition of viral DNA polymerase and modulation of host antiviral pathways, including NFKB1. Collectively, these findings indicate that these edible mushrooms serve as a valuable starting point for discovering novel anti-HSV agents.

## Figures and Tables

**Table 1:** Bioactive compound contents and antioxidant activities of mushroom extracts.

Bioactive Compound	Extract					
	LE_F	LE_D	HM_F	HM_D	PE_F	PE_D
Total polysaccharide (mg/g)	126.55 ± 0.22 ^a^	138.35 ± 3.29 ^b^	77.91 ± 1.87 ^e^	65.82 ± 0.87 ^f^	94.03 ± 1.56 ^d^	103.91 ± 1.54 ^c^
TPC (mg/g)	0.04 ± 0.01 ^a^	0.06 ± 0.04 ^a^	0.02 ± 0.03 ^a^	0.01 ± 0.05 ^a^	0.04 ± 0.03 ^a^	0.04 ± 0.01 ^a^
Terpenoid (mg/g)	6.03 ± 0.40 ^a^	6.70 ± 0.70 ^a^	2.00 ± 0.43 ^c^	1.91 ± 0.32 ^c^	2.80 ± 0.26 ^ab^	3.80 ± 0.26 ^b^
Total glucan (% *w*/*w*)	9.92 ± 0.44 ^ab^	10.43 ± 0.16 ^a^	9.71 ± 0.12 ^b^	10.03 ± 0.15 ^ab^	9.19 ± 0.02 ^c^	9.64 ± 0.02 ^b^
α-glucan (% *w*/*w*)	1.03 ± 0.08 ^a^	0.89 ± 0.07 ^a^	0.97 ± 0.05 ^a^	0.92 ± 0.04 ^a^	1.02 ± 0.13 ^a^	0.95 ± 0.07 ^a^
β-glucan (% *w*/*w*)	8.89 ± 0.47 ^ab^	9.54 ± 0.23 ^a^	8.73 ± 0.12 ^bc^	9.10 ± 0.18 ^ab^	8.16 ± 0.14 ^c^	8.68 ± 0.04 ^bc^
Total protein (mg/g)	14.65 ± 2.03 ^ac^	17.50 ± 0.64 ^a^	6.34 ± 4.80 ^c^	6.42 ± 0.52 ^c^	6.50 ± 1.32 ^c^	9.16 ± 3.48 ^bc^
Antioxidant activity						
DPPH (mg GAE/g)	0.07 ± 0.00 ^e^	0.99 ± 0.01 ^a^	0.02 ± 0.00 ^d^	0.10 ± 0.00 ^b^	0.01 ± 0.00 ^c^	0.02 ± 0.00 ^d^
FRAP (mg GAE/g)	0.17 ± 0.00 ^c^	3.11 ± 0.00 ^a^	0.06 ± 0.00 ^e^	0.60 ± 0.00 ^b^	0.07 ± 0.00 ^d^	0.05 ± 0.00 ^f^

Note: Data are presented as mean ± standard deviation (SD). Different superscript letters within the same column indicate statistically significant difference (*p* < 0.05) as determined by one-way ANOVA with Tukey’s multiple comparison test.

**Table 2:** Putative annotation of the top four metabolites with the highest peak area detected in positive-ion mode across six mushroom extracts using ESI-QTOF-MS analysis.

Extract	Positive-Ion Mode				
	Compound	RT (min)	(*m/z*)^+1^	Formula	Intensity (×10^6^)
LE_F	Eritanidine	2.81	254.08	C_9_H_11_N_5_O_4_	2.89
	γ-Glutamylleucine	8.67	261.14	C_11_H_20_N_2_O_5_	2.30
	5′-Methylthioadenosine	8.38	298.09	C_11_H_15_N_5_O_3_S	2.20
	Tetraethylthiuram disulfide	8.14	297.05	C_10_H_20_N_2_S_4_	2.02
LE_D	2-Methylbutyroylcarnitine	9.17	246.17	C_12_H_23_NO_4_	2.27
	Eritanidine	2.81	254.08	C_9_H_11_N_5_O_4_	1.98
	Indoline	7.47	120.08	C_8_H_9_N	1.56
	p-Tolyldiethanolamine	13.45	196.13	C_11_H_17_NO_2_	1.30
HM_F	Indoline	7.47	120.08	C_8_H_9_N	2.48
	3-Indoleacrylic acid	8.54	188.07	C_11_H_9_NO_2_	1.70
	Betaine	1.74	118.08	C_5_H_11_NO_2_	1.48
	Acetyl-L-carnitine	2.61	204.12	C_9_H_17_NO_4_	1.23
HM_D	*N*-acetyl-2-phenylethylamine	11.44	164.10	C_10_H_13_NO	2.40
	Nicotinic acid	2.68	124.03	C_6_H_5_NO_2_	2.07
	Styrene	7.95	105.07	C_8_H_8_	2.04
	Phytosphingosine	14.42	318.30	C_18_H_39_NO_3_	1.41
PE_F	5′-Methylthioadenosine	8.38	298.01	C_11_H_15_N_5_O_3_S	3.04
	Indoline	7.47	120.08	C_8_H_9_N	2.46
	4-Guanidinobutyric acid	2.44	146.09	C_5_H_11_N_3_O_2_	2.17
	3-Indoleacrylic acid	8.54	188.07	C_11_H_9_NO_2_	1.45
PE_D	*N*-acetyl-2-phenylethylamine	11.44	164.10	C_10_H_13_NO	3.32
	Phytosphingosine	14.42	318.30	C_18_H_39_NO_3_	2.46
	Indoline	7.47	120.08	C_8_H_9_N	2.07
	Tetramethylpyrazine	9.24	137.10	C_8_H_12_N_2_	1.65

**Table 3:** Putative annotation of the top four metabolites with the highest peak area detected in negative-ion mode across six mushroom extracts using ESI-QTOF-MS analysis.

Extract	Negative-Ion Mode				
	Compound	RT (min)	(*m/z*)^−1^	Formula	Intensity (×10^6^)
LE_F	Malic acid	2.01	133.01	C_4_H_6_O_5_	1.38
	Mannitol	1.67	181.06	C_6_H_14_O_6_	0.67
	*N*-acetylglutamic acid	3.88	188.05	C_7_H_11_NO_5_	0.43
LE_D	Malic acid	2.01	133.01	C_4_H_6_O_5_	1.48
	Pyroglutamic acid	3.40	128.03	C_5_H_7_NO_3_	1.03
	Mannitol	1.67	181.06	C_6_H_14_O_6_	0.76
HM_F	Malic acid	2.01	133.01	C_4_H_6_O_5_	1.59
	Pyroglutamic acid	3.40	128.03	C_5_H_7_NO_3_	0.75
	Xanthin	3.90	151.02	C_5_H_4_N_4_O_2_	0.43
HM_D	Phenyllactic acid	10.71	165.05	C_9_H_10_O_3_	0.97
	Acetylleucine	10.22	172.09	C_8_H_15_NO_3_	0.71
	Hydroxyisocaproic acid	10.14	131.06	C_6_H_12_O_3_	0.70
PE_F	Malic acid	2.01	133.01	C_4_H_6_O_5_	1.33
	Pyroglutamic acid	3.40	128.03	C_5_H_7_NO_3_	0.69
	Citric acid	3.2	191.01	C_6_H_8_O_7_	0.44
PE_D	Phenyllactic acid	10.71	165.05	C_9_H_10_O_3_	0.93
	Acetylleucine	10.22	172.09	C_8_H_15_NO_3_	0.83
	Hydroxyphenyllactic acid	8.76	181.04	C_9_H_10_O_4_	0.68

**Table 4:** CC_50_, IC_50_ and SI values of mushroom extracts against HSV-1 infection.

Extract	CC_50_ (mg/mL)	Anti-Viral Activity at Post-Entry Step			
		HSV-1_WT		HSV-1_Dxpiii	
		IC_50_ (mg/mL)	SI	IC_50_ (mg/mL)	SI
LE_F	29.07 ± 0.23 ^a^	6.06 ± 0.96 ^bcd^	4.80	1.60 ± 0.41 ^a^	18.17
HM_F	28.63 ± 0.42 ^a^	3.76 ± 1.72 ^abcd^	7.61	2.86 ± 0.89 ^ab^	10.01
PE_F	27.81 ± 0.10 ^a^	7.45 ± 0.50 ^cd^	3.73	6.14 ± 0.22 ^c^	4.53
LE_D	29.37 ± 0.39 ^a^	1.98 ± 0.72 ^abc^	14.83	2.14 ± 0.36 ^ab^	13.72
HM_D	28.95 ± 0.76 ^a^	1.61 ± 1.06 ^ab^	17.98	2.78 ± 0.34 ^ab^	10.41
PE_D	36.80 ± 2.08 ^b^	9.55 ± 4.96 ^d^	3.85	3.45 ± 0.65 ^b^	10.67
ACV	29.74 ± 2.17 ^a^	0.04 ± 0.01 ^a^	743.5	2.79 ± 0.37 ^ab^	10.66

Note: SI is calculated by cytotoxic concentration at 50% (CC_50_)/IC_50_. Data are presented as mean ± SD. Different superscript letters within the same column indicate statistically significant difference (*p* < 0.05) as determined by one-way ANOVA with Tukey’s multiple comparison test.

**Table 5:** Prediction of the candidate ligands on drug-likeness and toxicological properties.

Compound	Physicochemical Property					Lipophilicity and Water Solubility		Drug-Likeness		Predicted Toxicology
	MW g/mol (<500)	HBA (<10)	HBD (<5)	RB (<10)	TPSA (Å^2^) (<140)	LogP_o/w_ (<5)	LogS_ESOL_ log_10_ (mol/L) (>−4)	Bioavailability (>0.17)	Lipinski Criteria	ProTOX3.0 (>Class 4)
ACV	225.20	5	3	4	119.05	−0.99	−0.41	0.55	Yes	Class 5
ACL	203.24	4	0	6	66.43	−2.22	−0.53	0.55	Yes	Class 4
ATL	173.21	3	2	5	66.40	0.66	−0.91	0.85	Yes	Class 6
CTA	192.12	7	4	5	132.13	−1.51	0.38	0.56	Yes	Class 3
ETD	253.21	7	4	4	147.38	−1.43	−0.36	0.56	Yes	Class 4
GBA	145.16	2	3	4	106.14	−1.33	0.45	0.55	Yes	Class 6
GGL	260.29	6	4	9	129.72	−0.89	0.68	0.56	Yes	Class 5
HPL	182.17	4	3	2	77.76	0.46	−1.46	0.56	Yes	Class 4
IAL	187.19	2	2	2	53.09	1.81	−2.54	0.85	Yes	Class 5
MAS	297.33	6	3	3	144.61	−0.58	−1.65	0.55	Yes	Class 4
MBC	245.32	4	0	8	66.43	−1.30	−1.54	0.55	Yes	Class 3
NGA	189.17	5	3	6	103.70	−0.67	0.53	0.56	Yes	Class 6
PLA	166.17	3	2	3	57.53	1.02	−1.75	0.85	Yes	Class 4

Note: The symbols presented are MW—molecular weight (threshold ˂ 500 Da); HBA—hydrogen-bond acceptors (threshold ˂ 10); HBD—hydrogen-bond donors (threshold ˂ 5); RB—rotatable bonds (threshold ˂ 10); TPSA—topological polar surface area (threshold ˂ 140 Å^2^); LogP_o/w_—octanol-water partition coefficient (threshold ˂ 10); LogS_ESOL_—aqueous solubility estimated by the ESOL method, expressed as log_10_ (mol/L), where values > −4 indicate good solubility; Lipinski criteria—rule of five for drug-likeness (MW < 500, LogP < 5, HBD < 5, HBA < 10); ProTOX3.0—predicted toxicity class, where Class 1 is the most toxic, and Class 6 is non-toxic; values > Class 4 indicate relatively low acute toxicity.

## Data Availability

The data that support the findings of this study are available from the corresponding author, J.C., upon reasonable request.

## Supplementary Materials

The following supporting information can be downloaded at: https://www.mdpi.com/article/10.3390/molecules31122091/s1, Figure S1: Chromatograms of ESI-QTOF-MS from LE_F, LE_D, HM_F, HM_D, PE_F and PE_D extracts; Figure S2: The top 50 significant features from untargeted metabolomic profiles of LE, HM, and PE extracts in positive- and negative-ion modes; Figure S3: Plaque formation in LE_F, LE_D, HM_F, HM_D, LE_F and LE_D-treated HSV-1_WT and HSV-1_dxpiii-infected Vero cells at the pre-entry step; Figure S4: Plaque formation in LE_F, LE_D, HM_F, HM_D, LE_F and LE_D-treated HSV-1_WT and HSV-1_dxpiii-infected Vero cells at the post-entry step; Figure S5: Cytotoxicity and plaque inhibition assay of acyclovir; Figure S6: Effect of mushroom extracts on cellular *IL6*, *STAT3* and *NFKB1*, and HSV-1 *UL30* and *US6* mRNA expression in HSV-1 infected CaSki cells; Figure S7: Effect of mushroom extracts on cellular *IL6*, *STAT3* and *NFKB1* mRNA expression in uninfected CaSki and Vero cells; Figure S8: The candidate ligands from the six mushroom extracts docking with HSV-1 DNA polymerase (PDB ID: 8V1T); Figure S9: The putative protein targets and biological functions of the 12 candidate ligands; Figure S10: Fruiting bodies of medicinal-edible mushrooms *Lentinula edodes*, *Hypsizygus marmoreus*, and *Pleurotus eryngii*; Figure S11: Titer of HSV-1 propagation in Vero cell line; Table S1: Computational-based prediction of the positive-candidate ligands from edible mushrooms with HSV-1 DNA polymerase (PDB: 8V1T); Table S2: Computational-based prediction of the negative-candidate ligands from edible mushrooms with HSV-1 DNA polymerase (PDB: 8V1T); Table S3: Primer sequences.; Table S4: The exact *p*-value of all comparisons. File S1: ESI-QTOF-MS result.

## Abbreviations

The following abbreviations are used in this manuscript:

ACL	Acetyl-L-carnitine
ATL	Acetylleucine
BP	Biological process
CC	Cellular component
CC_50_	Cytotoxic concentration at 50%
CMC	Carboxymethyl cellulose
CTA	Citric acid
Dex	Dextran
DPPH	2,2-diphenyl-1-picrylhydrazyl
ESI-QTOF-MS	Electrospray ionization–quadrupole time-of-flight mass spectrometry
ETD	Eritanidine
FRAP	Ferric reducing antioxidant power
GAE	Gallic acid equivalent
GBA	4-Guanidinobutyric acid
GGL	γ-Glutamylleucine
GO	Gene Ontology
GOPOD	Glucose oxidase-peroxidase
HBA	Hydrogen-bond acceptors
HBD	Hydrogen-bond donors
HM	*Hypsizygus marmoreus*
HM_F	Extract derived from fresh *Hypsizygus marmoreus* fruiting body
HM_D	Extract derived from dried *Hypsizygus marmoreus* fruiting body
HPL	Hydroxyphenyllactic acid
HSV-1_dxpiii	Herpes simplex virus type 1, dxpIII strain
HSV-1_WT	Herpes simplex virus type 1, wild type
IAL	3-Indoleacrylic acid
IC_50_	Inhibitory concentration at 50%
KEGG	Kyoto Encyclopedia of Genes and Genomes
LC-MS/MS	Liquid chromatography-tandem mass spectrometry
LE	*Lentinula edodes*
LE_F	Extract derived from fresh *Lentinula edodes* fruiting body
LE_D	Extract derived from dried *Lentinula edodes* fruiting body
MAS	5′-Methylthioadenosine
MBC	2-Methylbutyroylcarnitine
MCC	Maximal Clique Centrality
MF	Molecular function
MOI	Multiplicity of infection
MONA	MassBank of North America
MW	Molecular weight
NGA	N-acetylglutamic acid
PCA	Principle component analysis
PE	*Pleurotus eryngi*
PE_F	Extract derived from fresh *Pleurotus eryngi* fruiting body
PE_D	Extract derived from dried *Pleurotus eryngi* fruiting body
PLA	Phenyllactic acid
PPI	Protein–protein interaction
RB	Rotatable bonds
SD	Standard deviation
SI	Selective index
TPSA	Topological polar surface area
