# Supplementary material for: Metabolomic Profiles and Anti-Herpes Simplex Virus (Wild-Type and Drug-Resistant) Properties of Water-Based Extracts of Lentinula edodes, Hypsizygus marmoreus and Pleurotus eryngii
Source: Molecules. 2026 Jun 14;31(12):2091. doi: 10.3390/molecules31122091 (PMC13304720; doi:10.3390/molecules31122091)
Supplement: Supplementary file 1 [file molecules-31-02091-s001.zip › Supplementary data.pdf]

Supplementary data

Metabolomic Profiles and Anti-Herpes Simplex Virus (Wild-Type and Drug-Resistant) Properties of Water-Based Extracts of *Lentinula edodes*, *Hypsizygus marmoreus* and *Pleurotus eryngii*

Chaleampol Loymunkong[1], Tipaya Ekalaksananan[2,3], Chamsai Pientong[2,3], Yaovapa Aramsirirujiwet[1] and Jureeporn Chuerduangphui[1,*]

[1]Department of Microbiology, Faculty of Science, Kasetsart University, Bangkok, Thailand.

[2]Department of Microbiology, Faculty of Medicine, Khon Kaen University, Khon Kaen 40002, Thailand.
[3]HPV & EBV and Carcinogenesis Research Group, Khon Kaen University, Khon Kaen 40002, Thailand.
* Corresponding author

Email: fscijoc@ku.ac.th

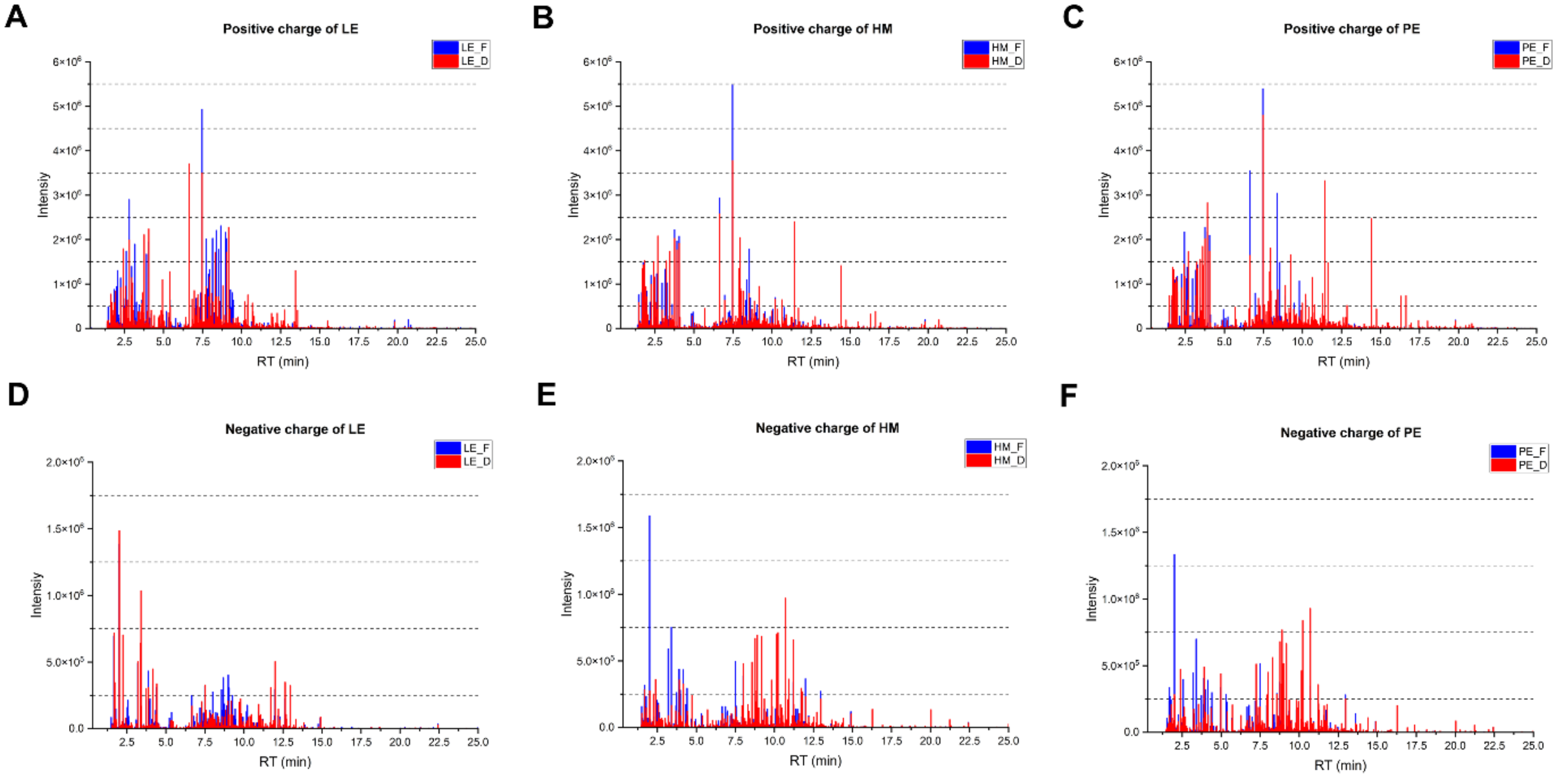

Figure S1. Chromatograms of ESI-QTOF-MS from LE_F, LE_D, HM_F, HM_D, PE_F and PE_D extracts. (**A-C**) Positive-ion modes of *L. edodes*, *H. marmoreus* and *P. eryngii* extracts, respectively. (**D-F**) Negative-ion modes of *L. edodes*, *H. marmoreus* and *P. eryngii* extracts, respectively. The data are shown in intensity of ion detection and retention time (RT) in unit of minute (min).

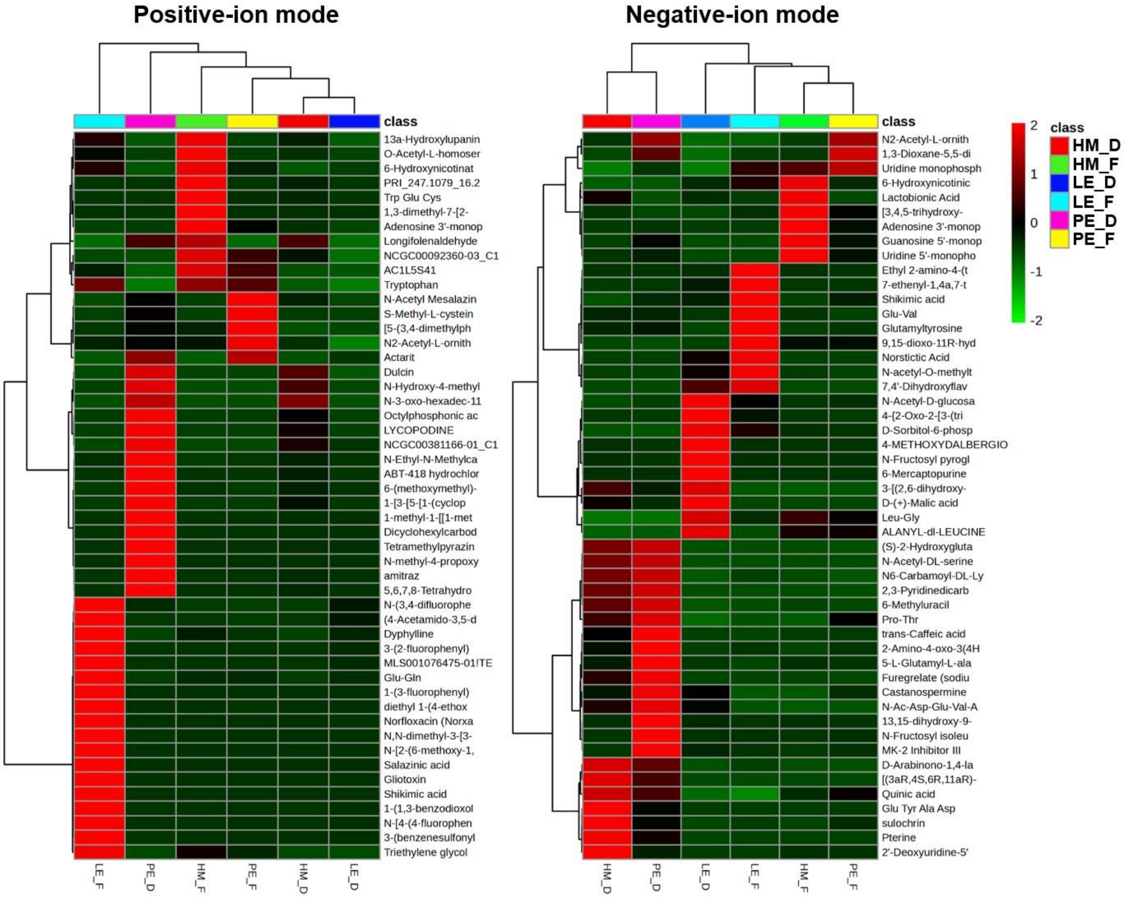

Figure S2. The top 50 significant features from untargeted metabolomic profiles of LE, HM, and PE extracts in positive- and negative-ion modes. Heat maps show the top 50 significant features (FDR < 0.05) of six extracts in positive- and negative-ion modes. Red and green colors represent high and low average intensity, respectively.

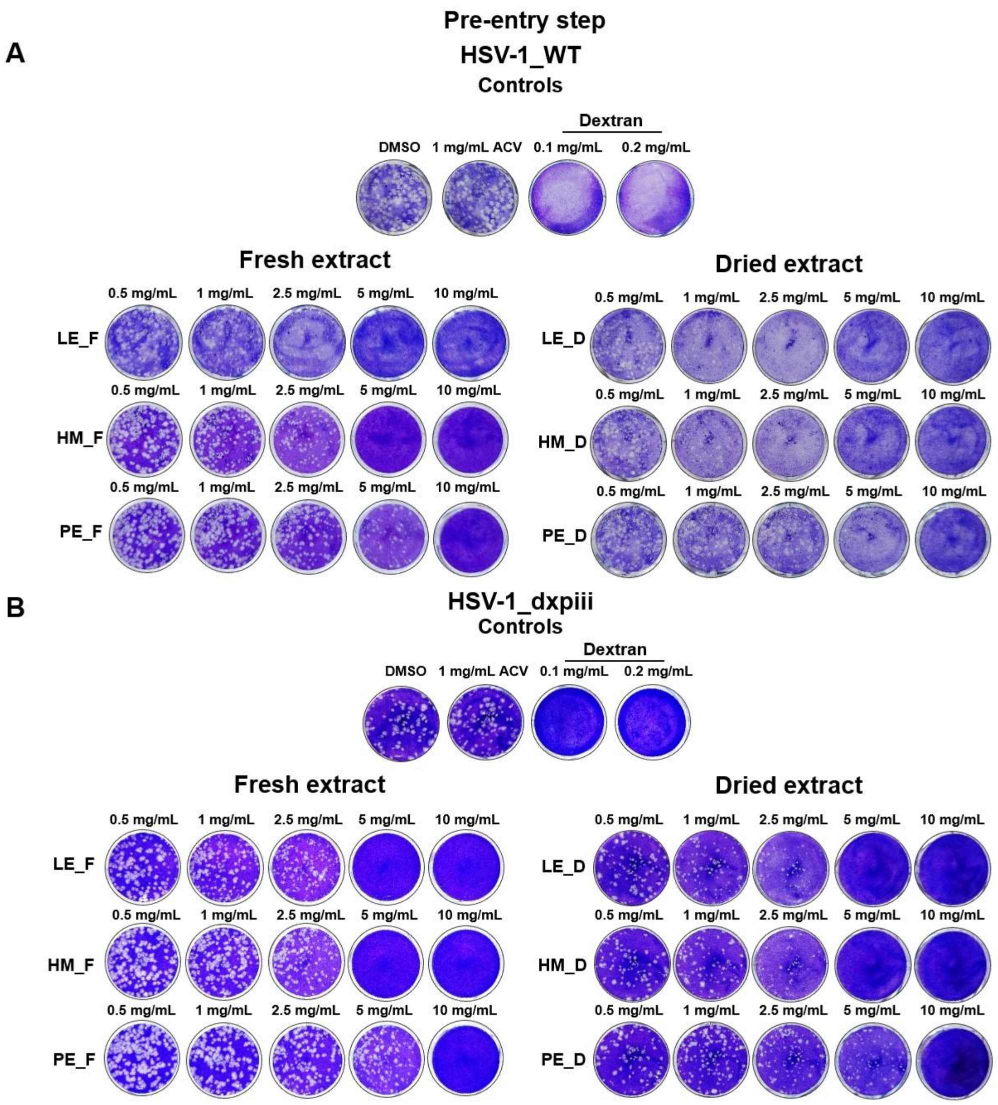

Figure S3. Plaque formation in LE_F, LE_D, HM_F, HM_D, LE_F and LE_D-treated HSV-1_WT and HSV-1_dxpiii-infected Vero cells at the pre-entry step. Plaque formation of fresh and dried extracts treatment in (**A**) HSV-1_WT and (**B**) HSV-1_dpxiii. Dextran (0.1 mg/mL and 0.2 mg/mL) and acyclovir (ACV) act as positive control.

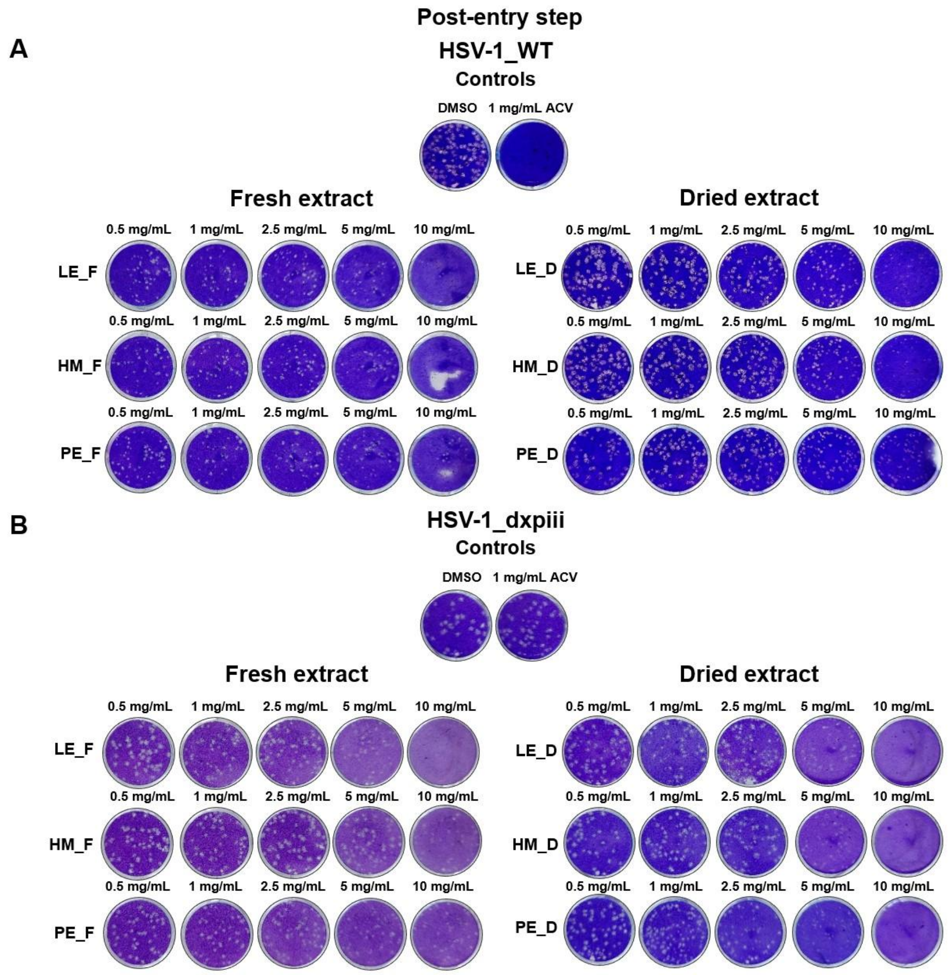

Figure S4. Plaque formation in LE_F, LE_D, HM_F, HM_D, LE_F and LE_D-treated HSV-1_WT and HSV-1_dxpiii-infected Vero cells at the post-entry step. Plaque formation of fresh and dried extracts treatment in (**A**) HSV-1_WT and (**B**) HSV-1_dpxiii. ACV acts as positive control.

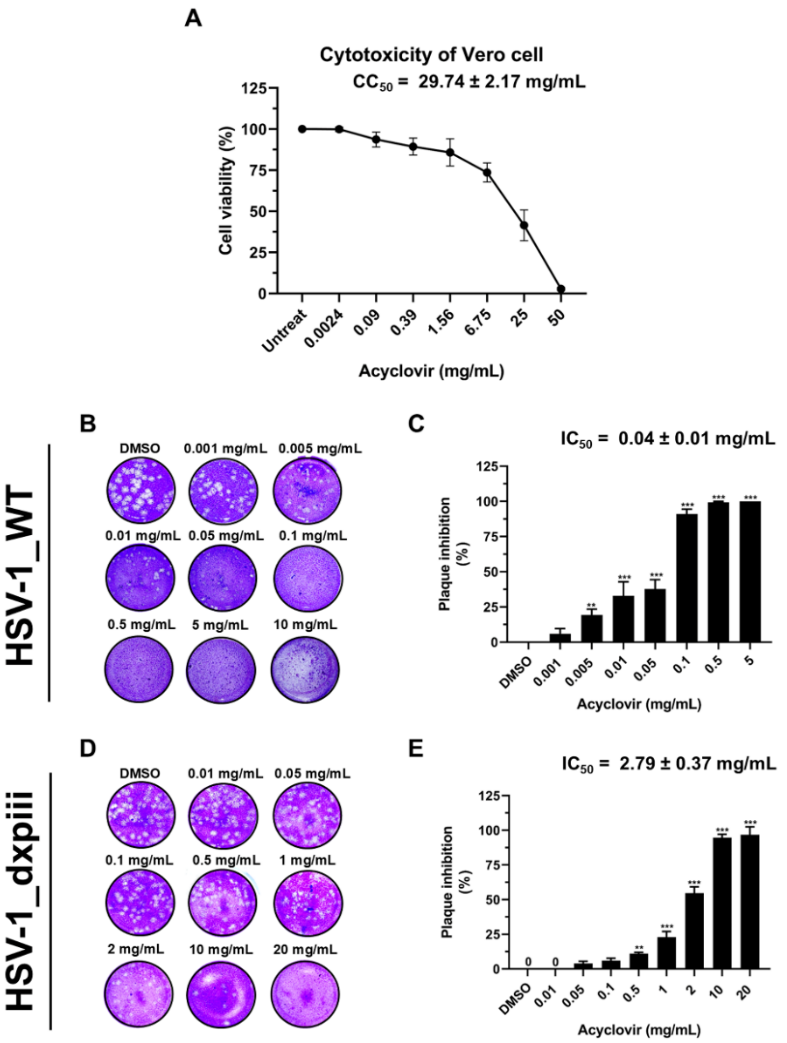

Figure S5. Cytotoxicity and plaque inhibition assay of acyclovir. (**A**) Vero cells were treated with the indicated concentration of acyclovir and then incubated for 72 h. The MTT assay were carried out to determine CC_{50}. (**B,D**) Plaque staining and (**C,E**) IC_{50} of (**B,C**) HSV-1_WT and (**D,E**) HSV-1_dxpiii infections. The data are shown as the mean ± SD (n=3). The significance is shown in (**), and (***) for $p < 0.01$ and $p < 0.001$, respectively.

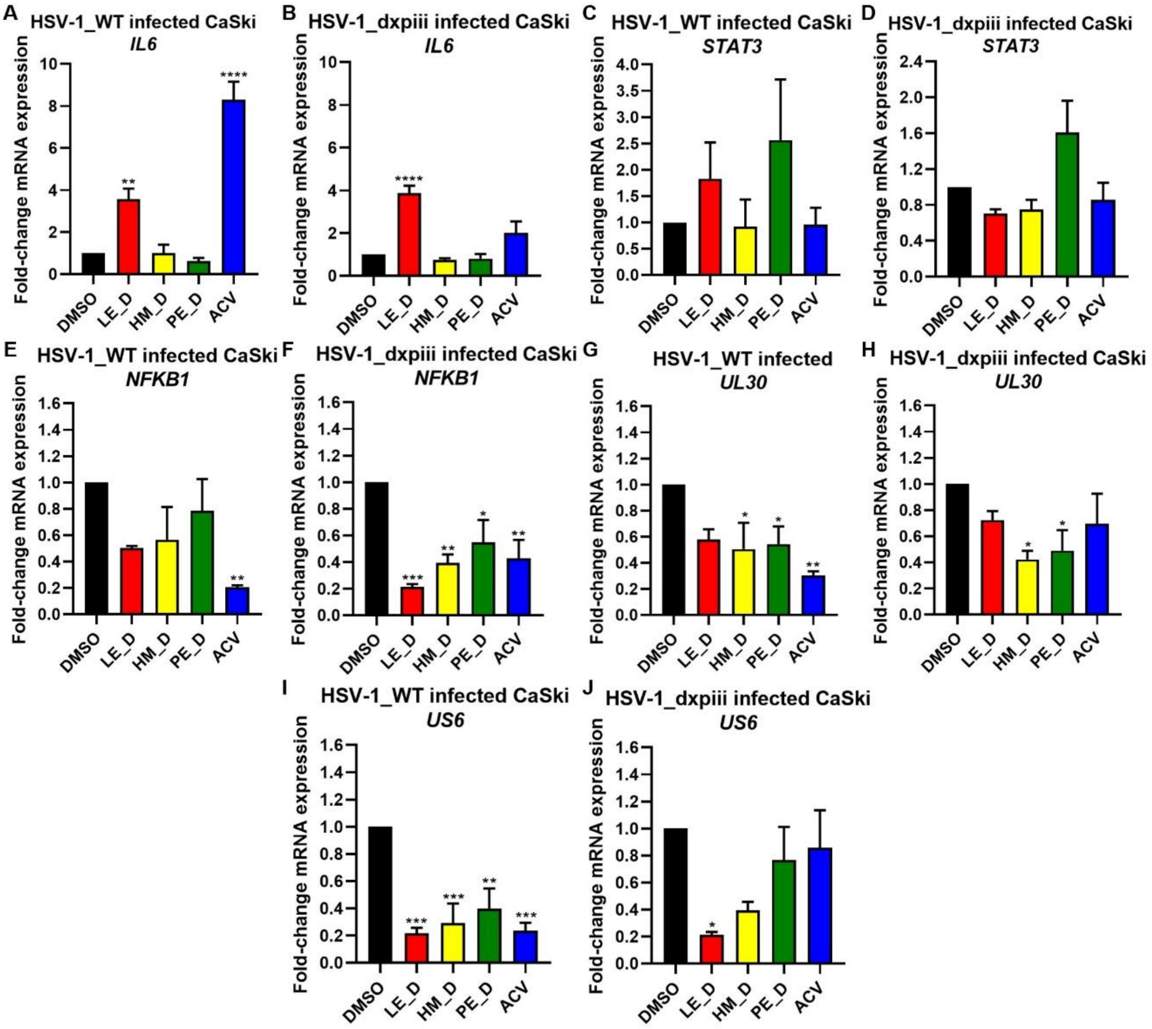

Figure S6. Effect of mushroom extracts on cellular *IL6*, *STAT3* and *NFKB1*, and HSV-1 *UL30* and *US6* mRNA expression in HSV-1 infected CaSki cells. (**A**,**B**) *IL6*, (**C**,**D**) *STAT3*, (**E**,**F**) *NFKB1*, (**G**,**H**) *UL30* and (**I**,**J**) *US6* were detected for quantifying their mRNA expression in (**A**,**C**,**E**,**G**,**I**) HSV-1_WT and (**B**,**D**,**F**,**H**,**J**) HSV-1_dxpiii infected CaSki cells. ACV at 1 mg/mL and DMSO were used for the positive and negative controls, respectively. Data are shown as the means ± SD. The symbols *, **, ***, and **** indicate significant difference ($p < 0.05$, 0.01, 0.001, 0.0001, respectively).

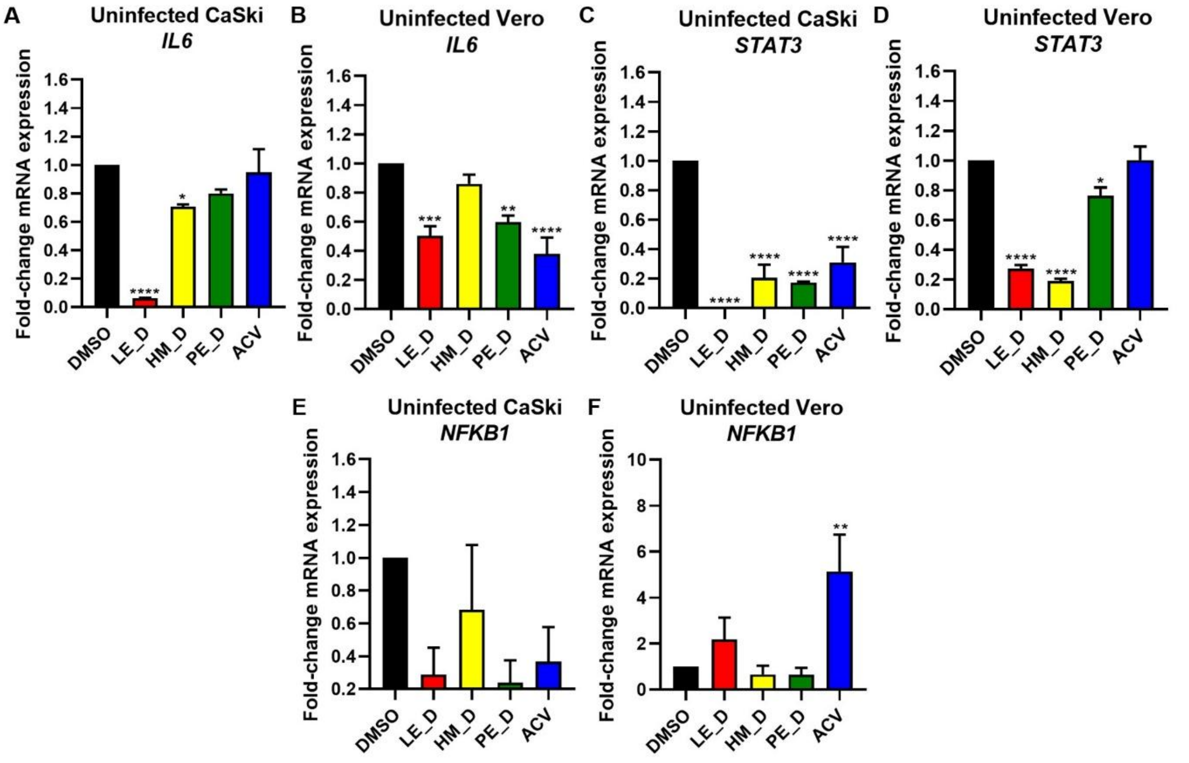

Figure S7. Effect of mushroom extracts on cellular *IL6*, *STAT3* and *NFKB1* mRNA expression in uninfected CaSki and Vero cells. (**A**,**B**) *IL6*, (**C**,**D**) *STAT3*, and (**E**,**F**) *NFKB1* were detected for quantifying their mRNA expression in uninfected (**A**,**C**,**E**) CaSki and (**B**,**D**,**F**) Vero cells. ACV at 1 mg/mL and DMSO were used for the positive and negative controls, respectively. Data are shown as the means ± SD. The symbols *, **, ***, and **** indicate significant difference ($p < 0.05$, 0.01, 0.001, 0.0001, respectively).

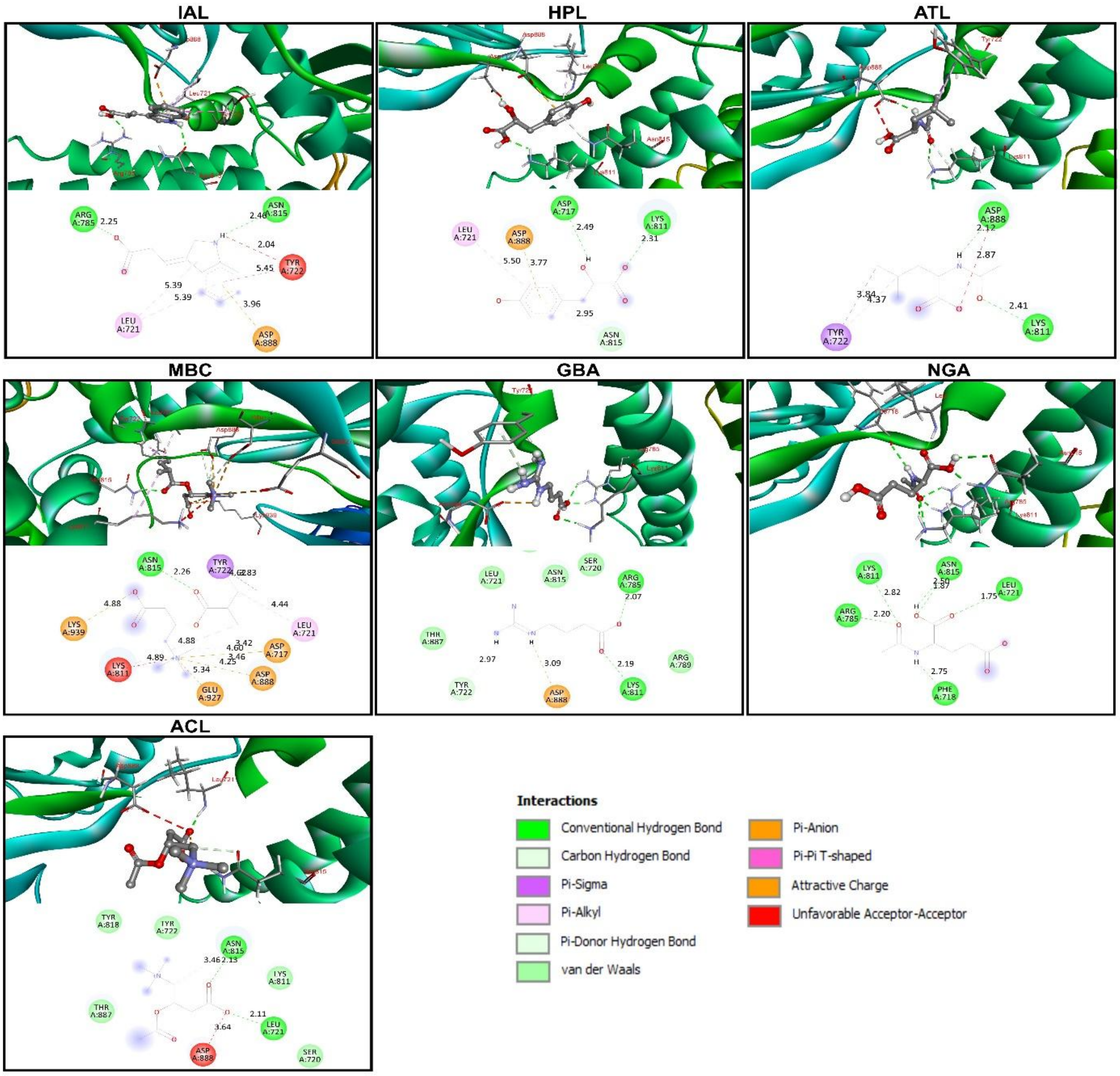

Figure S8. The candidate ligands from the six mushroom extracts docking with HSV-1 DNA polymerase (PDB ID: 8V1T). The grid center (x = 147.2865, y = 145.1162, and z = 123.7358 Å^3) and the grid box dimension (x = 20, y = 22, and z = 38 Å^3) were fixed in the active site to analyze the interaction between ligands and receptor. The interaction of ligands with the receptor are shown in the 2D and 3D structures. The indicated interactions are represented in the different color box as below. ΔG affinity binding of ligands with receptors are indoacylic acid (IAL) (6.39 kcal/mol), hydroxyphenylactic acid (HPL) (-6.05 kcal/mol), acetylleucine (ATL) (-5.48 kcal/mol), methylbutyroylcarnitine (MBC) (-5.40 kcal/mol), guanidinobutyric acid (GBA) (-5.39 kcal/mol), N-acetyl glutamic acid NGA (-5.28 kcal/mol), and acetyl-L-carnitine (ACL) (-5.19 kcal/mol).

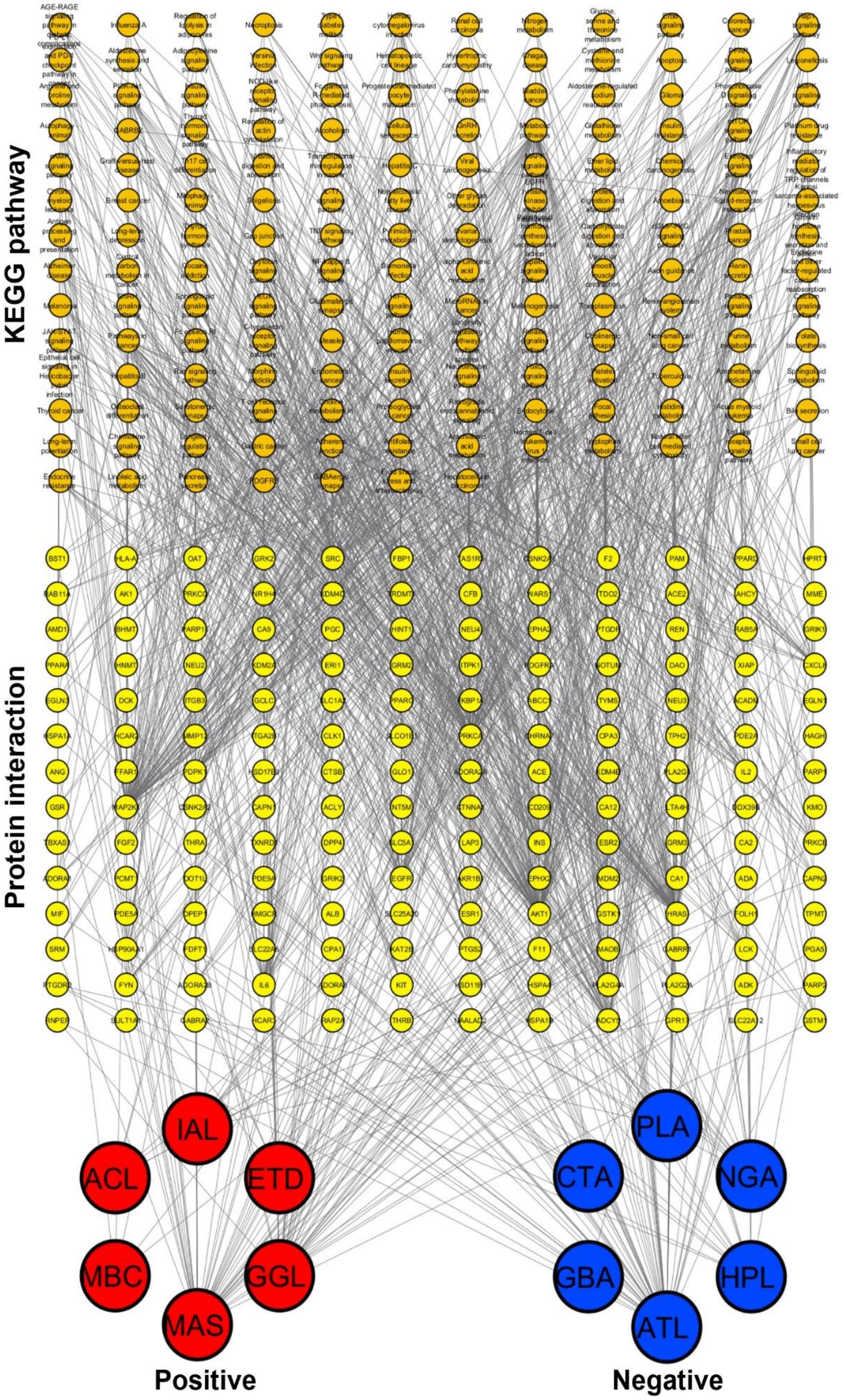

Figure S9. The putative protein targets and biological functions of the 12 candidate ligands. Compound-protein-pathway interaction network showing the relationships between positive- (red) and negative- (blue) candidate compounds, their interacting target proteins (yellow), and KEGG-enriched pathways (orange).

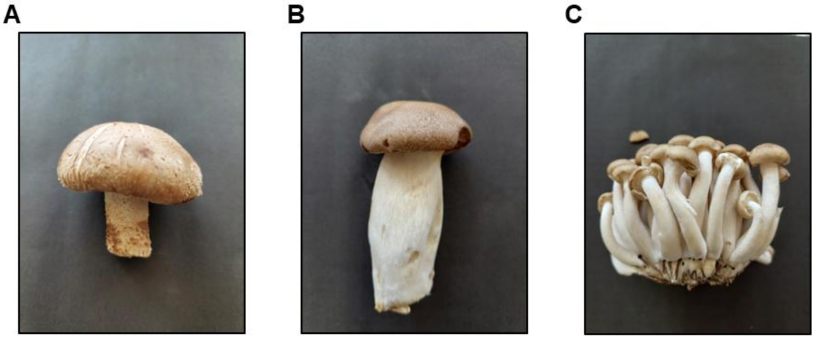

Figure S10. Fruiting bodies of medicinal-edible mushrooms *Lentinula edodes*, *Hypsizygus marmoreus*, and *Pleurotus eryngii*. (**A**) *Lentinula edodes*, (**B**) *Hypsizygus marmoreus*, (**C**) *Pleurotus eryngii* were used in the present study.

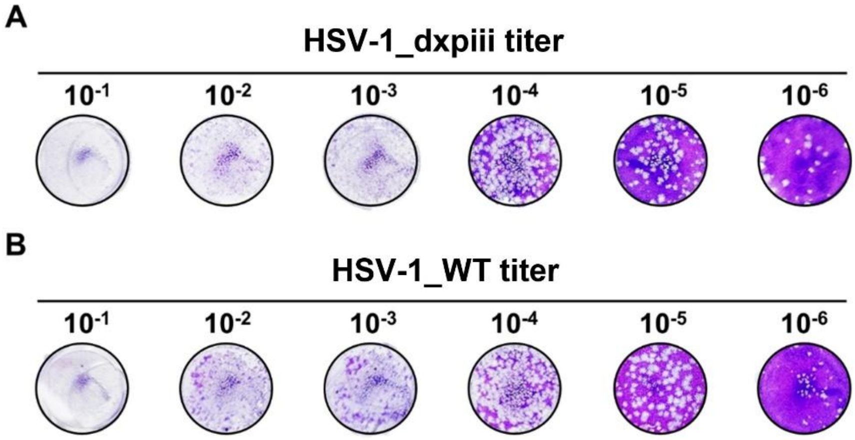

Figure S11. Titer of HSV-1 propagation in Vero cell line. The preparation of the virus from 10-folded dilution for (**A**) HSV1_dxpiii and (**B**) HSV1-WT strains. The viruses were infected into the cells for 2 h. and then, unbound viruses were removed. The virus-infected cells were incubated with 0.5% CMC in complete media for 72 h. The plaques were stained and counted. The dilution at 10^{-5} was selected for the plaque assay in both strains and the plaque numbers were counted.

Table S1 Computational-based prediction of the positive-candidate ligands from edible mushrooms with HSV-1 DNA polymerase (PDB: 8V1T).

Compound	Affinity binding (kcal/mol)	RMSD (Å)
Eritanidine	−7.95	0.00
γ-Glutamylleucine	−6.86	0.00
5'-Methylthioadenosine	−7.08	1.58
Tetraethylthiuram disulfide	−4.51	1.24
2-Methylbutyroylcarnitine	−5.40	1.57
Indoline	−4.74	1.61
p-Tolyldiethanolamine	−4.93	0.11
3-Indoleacrylic acid	−6.39	1.49
Betaine	−3.54	1.72
Acetyl-L-carnitine	−5.19	0.15
N-acetyl-2-phenylethylamine	−4.16	1.19
Nicotinic acid	−4.61	1.68
Styrene	−4.29	0.80
Phytosphingosine	−4.36	1.97
4-Guanidinobutyric acid	−5.39	0.00
N-acetyl-2-phenylethylamine	−4.73	2.27
Tetramethylpyrazine	−4.42	0.12

Table S2 Computational-based prediction of the negative-candidate ligands from edible mushrooms with HSV-1 DNA polymerase (PDB: 8V1T).

Compound	Affinity binding (kcal/mol)	RMSD (Å)
Malic acid	−4.68	1.71
Mannitol	−4.69	1.74
N-acetylglutamic acid	−5.57	0.00
Malic acid	−4.10	0.69
Pyroglutamic acid	−4.61	1.77
Xanthin	−4. 96	1.93
Acetylleucine	−5.48	0.00
Hydroxyisocaproic acid	−4.96	1.22
Citric acid	−6.68	0.00
Phenyllactic acid	−6.52	0.00
Hydroxyphenyllactic acid	−6.05	0.00

Table S3 Primer sequences.

Gene	Primer	Sequence
UL30	Forward_UL30	5’ GTGTTGTGCCGCGGTCTCAC 3’
	Reverse_UL30	5’ GGTGAACGTCTTTTCGAACTC 3’
US6	Forward_US6	5’ AGCAGGGGTTAGGGAGTTGT 3’
	Reverse_US6	5’ CCATCTTGAGAGAGGCATCC 3’
NFKB1	Forward_NF-κB	5’ GAAATTCCTGATCCAGACAAAAAC 3’
	Reverse_NF-κB	5’ ATCACTTCAATGGCCTCTGTGTAG 3’
IL6	Forward_IL-6	5’ ATGTAGCCGCCCCACACAGA 3’
	Reverse_IL-6	5’ CATCCATCTTTTTCAGACAT3’
STAT3	Forward_STAT3	5’ TGTGCGTATGGGAACACCTA 3’
	Reverse_STAT3	5’ AGAAGGTCGTCTCCCCCTTA 3’
GAPDH	Forward_GAPDH	5’ TCATCAGCAATGCCTCCTGCA 3’
	Reverse_GAPDH	5’ TGGGTAGCAGTGATGGCA 3’

Table S4 The exact p-value of all comparisons.

Tukey's multiple comparisons test	Mean Diff.	95.00% CI of diff.	Summary	Adjusted P Value
Polysaccharide among six extracts				
LE_F vs. LE_D	-11.8	-16.80 to -6.801	****	<0.0001
LE_F vs. HM_F	48.64	43.64 to 53.64	****	<0.0001
LE_F vs. HM_D	60.73	55.73 to 65.73	****	<0.0001
LE_F vs. PE_F	32.52	27.52 to 37.52	****	<0.0001
LE_F vs. PE_D	22.64	17.64 to 27.64	****	<0.0001
LE_D vs. HM_F	60.44	55.44 to 65.44	****	<0.0001
LE_D vs. HM_D	72.53	67.53 to 77.53	****	<0.0001
LE_D vs. PE_F	44.32	39.32 to 49.32	****	<0.0001
LE_D vs. PE_D	34.44	29.44 to 39.44	****	<0.0001
HM_F vs. HM_D	12.09	7.091 to 17.09	****	<0.0001
HM_F vs. PE_F	-16.12	-21.12 to -11.12	****	<0.0001
HM_F vs. PE_D	-26	-31.00 to -21.00	****	<0.0001
HM_D vs. PE_F	-28.21	-33.21 to -23.21	****	<0.0001
HM_D vs. PE_D	-38.09	-43.09 to -33.09	****	<0.0001
PE_F vs. PE_D	-9.88	-14.88 to -4.881	***	0.0003
Total phenolic content among six extracts				
LE_F vs. LE_D	-0.02	-0.1074 to 0.06745	ns	0.968
LE_F vs. HM_F	0.02	-0.06745 to 0.1074	ns	0.968
LE_F vs. HM_D	0.03	-0.05745 to 0.1174	ns	0.8503
LE_F vs. PE_F	0	-0.08745 to 0.08745	ns	>0.9999
LE_F vs. PE_D	0	-0.08745 to 0.08745	ns	>0.9999
LE_D vs. HM_F	0.04	-0.04745 to 0.1274	ns	0.6499
LE_D vs. HM_D	0.05	-0.03745 to 0.1374	ns	0.4356
LE_D vs. PE_F	0.02	-0.06745 to 0.1074	ns	0.968
LE_D vs. PE_D	0.02	-0.06745 to 0.1074	ns	0.968
HM_F vs. HM_D	0.01	-0.07745 to 0.09745	ns	0.9986
HM_F vs. PE_F	-0.02	-0.1074 to 0.06745	ns	0.968
HM_F vs. PE_D	-0.02	-0.1074 to 0.06745	ns	0.968
HM_D vs. PE_F	-0.03	-0.1174 to 0.05745	ns	0.8503
HM_D vs. PE_D	-0.03	-0.1174 to 0.05745	ns	0.8503
PE_F vs. PE_D	0	-0.08745 to 0.08745	ns	>0.9999
Terpenoid among six extracts				
LE_F vs. LE_D	-0.67	-1.830 to 0.4895	ns	0.4251
LE_F vs. HM_F	4.03	2.870 to 5.190	****	<0.0001
LE_F vs. HM_D	4.12	2.960 to 5.280	****	<0.0001
LE_F vs. PE_F	3.23	2.070 to 4.390	****	<0.0001
LE_F vs. PE_D	2.23	1.070 to 3.390	***	0.0003
LE_D vs. HM_F	4.7	3.540 to 5.860	****	<0.0001
LE_D vs. HM_D	4.79	3.630 to 5.950	****	<0.0001
LE_D vs. PE_F	3.9	2.740 to 5.060	****	<0.0001
LE_D vs. PE_D	2.9	1.740 to 4.060	****	<0.0001
HM_F vs. HM_D	0.09	-1.070 to 1.250	ns	0.9998
HM_F vs. PE_F	-0.8	-1.960 to 0.3595	ns	0.2589
HM_F vs. PE_D	-1.8	-2.960 to -0.6405	**	0.0023
HM_D vs. PE_F	-0.89	-2.050 to 0.2695	ns	0.1764
HM_D vs. PE_D	-1.89	-3.050 to -0.7305	**	0.0015
PE_F vs. PE_D	-1	-2.160 to 0.1595	ns	0.107
Total protein among six extracts				
LE_F vs. LE_D	-2.85	-10.08 to 4.380	ns	0.7674
LE_F vs. HM_F	8.31	1.080 to 15.54	*	0.0214
LE_F vs. HM_D	8.23	1.000 to 15.46	*	0.0228
LE_F vs. PE_F	8.15	0.9204 to 15.38	*	0.0243
LE_F vs. PE_D	5.49	-1.740 to 12.72	ns	0.1839
LE_D vs. HM_F	11.16	3.930 to 18.39	**	0.0024
LE_D vs. HM_D	11.08	3.850 to 18.31	**	0.0026
LE_D vs. PE_F	11	3.770 to 18.23	**	0.0027

LE_D vs. PE_D	8.34	1.110 to 15.57	*	0.0209
HM_F vs. HM_D	-0.08	-7.310 to 7.150	ns	>0.9999
HM_F vs. PE_F	-0.16	-7.390 to 7.070	ns	>0.9999
HM_F vs. PE_D	-2.82	-10.05 to 4.410	ns	0.7747
HM_D vs. PE_F	-0.08	-7.310 to 7.150	ns	>0.9999
HM_D vs. PE_D	-2.74	-9.970 to 4.490	ns	0.7936
PE_F vs. PE_D	-2.66	-9.890 to 4.570	ns	0.8119
Total glucan among six extracts				
LE_F vs. LE_D	-0.51	-1.07881693794233 to 0.0588169379423295	ns	0.088857
LE_F vs. HM_F	0.21	-0.358816937942330 to 0.778816937942328	ns	0.80983
LE_F vs. HM_D	-0.11	-0.678816937942329 to 0.458816937942330	ns	0.984384
LE_F vs. PE_F	0.73	0.161183062057671 to 1.29881693794233	*	0.010057
LE_F vs. PE_D	0.28	-0.288816937942330 to 0.848816937942329	ns	0.582747
LE_D vs. HM_F	0.72	0.151183062057670 to 1.28881693794233	*	0.011103
LE_D vs. HM_D	0.4	-0.168816937942329 to 0.968816937942330	ns	0.242972
LE_D vs. PE_F	1.24	0.671183062057671 to 1.80881693794233	***	0.000104
LE_D vs. PE_D	0.79	0.221183062057670 to 1.35881693794233	**	0.005585
HM_F vs. HM_D	-0.32	-0.888816937942328 to 0.248816937942331	ns	0.451748
HM_F vs. PE_F	0.52	-0.0488169379423279 to 1.08881693794233	ns	0.080674
HM_F vs. PE_D	0.07	-0.498816937942329 to 0.638816937942330	ns	0.998036
HM_D vs. PE_F	0.84	0.271183062057671 to 1.40881693794233	**	0.00345
HM_D vs. PE_D	0.39	-0.178816937942330 to 0.958816937942328	ns	0.264287
PE_F vs. PE_D	-0.45	-1.01881693794233 to 0.118816937942328	ns	0.156237
LE_F vs. LE_D	-0.51	-1.07881693794233 to 0.0588169379423295	ns	0.088857
LE_F vs. HM_F	0.21	-0.358816937942330 to 0.778816937942328	ns	0.80983
LE_F vs. HM_D	-0.11	-0.678816937942329 to 0.458816937942330	ns	0.984384
Alpha-glucan among six extracts				
LE_F vs. LE_D	0.14	-0.0759483985846250 to 0.355948398584625	ns	0.314187
LE_F vs. HM_F	0.06	-0.155948398584625 to 0.275948398584625	ns	0.93005
LE_F vs. HM_D	0.11	-0.105948398584625 to 0.325948398584625	ns	0.549924
LE_F vs. PE_F	0.01	-0.205948398584625 to 0.225948398584625	ns	0.999983
LE_F vs. PE_D	0.08	-0.135948398584625 to 0.295948398584625	ns	0.807754
LE_D vs. HM_F	-0.08	-0.295948398584625 to 0.135948398584625	ns	0.807754
LE_D vs. HM_D	-0.03	-0.245948398584625 to 0.185948398584625	ns	0.996526
LE_D vs. PE_F	-0.13	-0.345948398584625 to 0.0859483985846250	ns	0.384745
LE_D vs. PE_D	-0.06	-0.275948398584625 to 0.155948398584625	ns	0.93005
HM_F vs. HM_D	0.05	-0.165948398584625 to 0.265948398584625	ns	0.966275
HM_F vs. PE_F	-0.05	-0.265948398584625 to 0.165948398584625	ns	0.966275
HM_F vs. PE_D	0.02	-0.195948398584625 to 0.235948398584625	ns	0.999498
HM_D vs. PE_F	-0.1	-0.315948398584625 to 0.115948398584625	ns	0.639061
HM_D vs. PE_D	-0.03	-0.245948398584625 to 0.185948398584625	ns	0.996526
PE_F vs. PE_D	0.07	-0.145948398584625 to 0.285948398584625	ns	0.876771
Beta-glucan among six extracts				
LE_F vs. LE_D	-0.65	-1.305 to 0.004583	ns	0.052
LE_F vs. HM_F	0.16	-0.4946 to 0.8146	ns	0.9579
LE_F vs. HM_D	-0.21	-0.8646 to 0.4446	ns	0.8812
LE_F vs. PE_F	0.73	0.07542 to 1.385	*	0.026
LE_F vs. PE_D	0.21	-0.4446 to 0.8646	ns	0.8812
LE_D vs. HM_F	0.81	0.1554 to 1.465	*	0.013
LE_D vs. HM_D	0.44	-0.2146 to 1.095	ns	0.2815
LE_D vs. PE_F	1.38	0.7254 to 2.035	***	0.0001
LE_D vs. PE_D	0.86	0.2054 to 1.515	**	0.0085
HM_F vs. HM_D	-0.37	-1.025 to 0.2846	ns	0.447
HM_F vs. PE_F	0.57	-0.08458 to 1.225	ns	0.1023
HM_F vs. PE_D	0.05	-0.6046 to 0.7046	ns	0.9998
HM_D vs. PE_F	0.94	0.2854 to 1.595	**	0.0043
HM_D vs. PE_D	0.42	-0.2346 to 1.075	ns	0.3238
PE_F vs. PE_D	-0.52	-1.175 to 0.1346	ns	0.1536
Activity of 2,2-diphenyl-1-picrylhydrazyl radical scavenging among six extracts				
LE_F vs. LE_D	-0.9198	-0.9264 to -0.9133	****	<0.0001
LE_F vs. HM_F	0.04689	0.04033 to 0.05345	****	<0.0001

LE_F vs. HM_D	-0.02904	-0.03560 to -0.02248	****	<0.0001
LE_F vs. PE_F	0.0635	0.05693 to 0.07006	****	<0.0001
LE_F vs. PE_D	0.04715	0.04058 to 0.05371	****	<0.0001
LE_D vs. HM_F	0.9667	0.9602 to 0.9733	****	<0.0001
LE_D vs. HM_D	0.8908	0.8842 to 0.8974	****	<0.0001
LE_D vs. PE_F	0.9833	0.9768 to 0.9899	****	<0.0001
LE_D vs. PE_D	0.967	0.9604 to 0.9735	****	<0.0001
HM_F vs. HM_D	-0.07593	-0.08249 to -0.06937	****	<0.0001
HM_F vs. PE_F	0.01661	0.01004 to 0.02317	****	<0.0001
HM_F vs. PE_D	0.000255	-0.006307 to 0.006817	ns	>0.9999
HM_D vs. PE_F	0.09254	0.08597 to 0.09910	****	<0.0001
HM_D vs. PE_D	0.07619	0.06962 to 0.08275	****	<0.0001
PE_F vs. PE_D	-0.01635	-0.02291 to -0.009788	****	<0.0001
Activity of ferric reducing antioxidant power among six extracts				
LE_F vs. LE_D	-2.94	-2.943 to -2.937	****	<0.0001
LE_F vs. HM_F	0.1156	0.1127 to 0.1184	****	<0.0001
LE_F vs. HM_D	-0.4231	-0.4259 to -0.4202	****	<0.0001
LE_F vs. PE_F	0.1028	0.09992 to 0.1056	****	<0.0001
LE_F vs. PE_D	0.1235	0.1207 to 0.1264	****	<0.0001
LE_D vs. HM_F	3.055	3.053 to 3.058	****	<0.0001
LE_D vs. HM_D	2.517	2.514 to 2.520	****	<0.0001
LE_D vs. PE_F	3.043	3.040 to 3.045	****	<0.0001
LE_D vs. PE_D	3.063	3.061 to 3.066	****	<0.0001
HM_F vs. HM_D	-0.5386	-0.5415 to -0.5358	****	<0.0001
HM_F vs. PE_F	-0.0128	-0.01564 to -0.009947	****	<0.0001
HM_F vs. PE_D	0.007965	0.005117 to 0.01081	****	<0.0001
HM_D vs. PE_F	0.5258	0.5230 to 0.5287	****	<0.0001
HM_D vs. PE_D	0.5466	0.5437 to 0.5494	****	<0.0001
PE_F vs. PE_D	0.02076	0.01791 to 0.02361	****	<0.0001
CC_{50} of the extract in Vero cells				
LE_F vs. LE_D	-0.3	-3.633 to 3.033	ns	>0.9999
LE_F vs. HM_F	0.44	-2.893 to 3.773	ns	0.9991
LE_F vs. HM_D	0.12	-3.213 to 3.453	ns	>0.9999
LE_F vs. PE_F	1.26	-2.073 to 4.593	ns	0.8453
LE_F vs. PE_D	-7.73	-11.06 to -4.397	****	<0.0001
LE_F vs. Acyclovir	-0.67	-4.003 to 2.663	ns	0.9913
LE_D vs. HM_F	0.74	-2.593 to 4.073	ns	0.9856
LE_D vs. HM_D	0.42	-2.913 to 3.753	ns	0.9993
LE_D vs. PE_F	1.56	-1.773 to 4.893	ns	0.6858
LE_D vs. PE_D	-7.43	-10.76 to -4.097	****	<0.0001
LE_D vs. Acyclovir	-0.37	-3.703 to 2.963	ns	0.9997
HM_F vs. HM_D	-0.32	-3.653 to 3.013	ns	0.9999
HM_F vs. PE_F	0.82	-2.513 to 4.153	ns	0.976
HM_F vs. PE_D	-8.17	-11.50 to -4.837	****	<0.0001
HM_F vs. Acyclovir	-1.11	-4.443 to 2.223	ns	0.9058
HM_D vs. PE_F	1.14	-2.193 to 4.473	ns	0.895
HM_D vs. PE_D	-7.85	-11.18 to -4.517	****	<0.0001
HM_D vs. Acyclovir	-0.79	-4.123 to 2.543	ns	0.98
PE_F vs. PE_D	-8.99	-12.32 to -5.657	****	<0.0001
PE_F vs. Acyclovir	-1.93	-5.263 to 1.403	ns	0.4677
PE_D vs. Acyclovir	7.06	3.727 to 10.39	****	<0.0001
IC_{50} of HSV-1_dxpiii				
LE_F vs. LE_D	4.08	-1.728 to 9.888	ns	0.2678
LE_F vs. HM_F	2.3	-3.508 to 8.108	ns	0.8169
LE_F vs. HM_D	4.45	-1.358 to 10.26	ns	0.1928
LE_F vs. PE_F	-1.39	-7.198 to 4.418	ns	0.979
LE_F vs. PE_D	-3.49	-9.298 to 2.318	ns	0.4275
LE_F vs. Acyclovir	6.02	0.2125 to 11.83	*	0.04
LE_D vs. HM_F	-1.78	-7.588 to 4.028	ns	0.9337
LE_D vs. HM_D	0.37	-5.438 to 6.178	ns	>0.9999
LE_D vs. PE_F	-5.47	-11.28 to 0.3375	ns	0.071

LE_D vs. PE_D	-7.57	-13.38 to -1.762	**	0.0076
LE_D vs. Acyclovir	1.94	-3.868 to 7.748	ns	0.9046
HM_F vs. HM_D	2.15	-3.658 to 7.958	ns	0.8569
HM_F vs. PE_F	-3.69	-9.498 to 2.118	ns	0.3681
HM_F vs. PE_D	-5.79	-11.60 to 0.01754	ns	0.0509
HM_F vs. Acyclovir	3.72	-2.088 to 9.528	ns	0.3596
HM_D vs. PE_F	-5.84	-11.65 to -0.03246	*	0.0483
HM_D vs. PE_D	-7.94	-13.75 to -2.132	**	0.0051
HM_D vs. Acyclovir	1.57	-4.238 to 7.378	ns	0.9624
PE_F vs. PE_D	-2.1	-7.908 to 3.708	ns	0.8692
PE_F vs. Acyclovir	7.41	1.602 to 13.22	**	0.0091
PE_D vs. Acyclovir	9.51	3.702 to 15.32	**	0.001
IC_{50} of HSV-1_dxpiii				
LE_F vs. LE_D	-0.54	-1.959 to 0.8789	ns	0.8414
LE_F vs. HM_F	-1.26	-2.679 to 0.1589	ns	0.0975
LE_F vs. HM_D	-1.18	-2.599 to 0.2389	ns	0.1347
LE_F vs. PE_F	-4.54	-5.959 to -3.121	****	<0.0001
LE_F vs. PE_D	-1.85	-3.269 to -0.4311	**	0.0076
LE_F vs. Acyclovir	-1.19	-2.609 to 0.2289	ns	0.1294
LE_D vs. HM_F	-0.72	-2.139 to 0.6989	ns	0.6077
LE_D vs. HM_D	-0.64	-2.059 to 0.7789	ns	0.7186
LE_D vs. PE_F	-4	-5.419 to -2.581	****	<0.0001
LE_D vs. PE_D	-1.31	-2.729 to 0.1089	ns	0.0793
LE_D vs. Acyclovir	-0.65	-2.069 to 0.7689	ns	0.7051
HM_F vs. HM_D	0.08	-1.339 to 1.499	ns	>0.9999
HM_F vs. PE_F	-3.28	-4.699 to -1.861	****	<0.0001
HM_F vs. PE_D	-0.59	-2.009 to 0.8289	ns	0.7833
HM_F vs. Acyclovir	0.07	-1.349 to 1.489	ns	>0.9999
HM_D vs. PE_F	-3.36	-4.779 to -1.941	****	<0.0001
HM_D vs. PE_D	-0.67	-2.089 to 0.7489	ns	0.6777
HM_D vs. Acyclovir	-0.01	-1.429 to 1.409	ns	>0.9999
PE_F vs. PE_D	2.69	1.271 to 4.109	***	0.0002
PE_F vs. Acyclovir	3.35	1.931 to 4.769	****	<0.0001
PE_D vs. Acyclovir	0.66	-0.7589 to 2.079	ns	0.6914
Dunnett's multiple comparisons test	Mean Diff.	95.00% CI of diff.	Summary	Adjusted P Value
Effect of fresh extracts against HSV-1_WT in pre-entry step				
DMSO vs. 0.5 LE_F	-21.6667	-26.6288291897375 to -16.7045041435958	****	<0.0001
DMSO vs. 1 LE_F	-34	-38.9621625230708 to -29.0378374769292	****	<0.0001
DMSO vs. 2.5 LE_F	-79.3333	-84.2954958564042 to -74.3711708102625	****	<0.0001
DMSO vs. 5 LE_F	-99.3333	-104.295495856404 to -94.3711708102625	****	<0.0001
DMSO vs. 10 LE_F	-99.3333	-104.295495856404 to -94.3711708102625	****	<0.0001
DMSO vs. 0.5 HM_F	-2	-6.96216252307083 to 2.96216252307083	ns	0.913369
DMSO vs. 1 HM_F	-26	-30.9621625230708 to -21.0378374769292	****	<0.0001
DMSO vs. 2.5 HM_F	-68.6667	-73.6288291897375 to -63.7045041435958	****	<0.0001
DMSO vs. 5 HM_F	-91.3333	-96.2954958564042 to -86.3711708102625	****	<0.0001
DMSO vs. 10 HM_F	-99.3333	-104.295495856404 to -94.3711708102625	****	<0.0001
DMSO vs. 0.5 PE_F	0	-4.96216252307083 to 4.96216252307083	ns	>0.9999
DMSO vs. 1 PE_F	-16	-20.9621625230708 to -11.0378374769292	****	<0.0001
DMSO vs. 2.5 PE_F	-31	-35.9621625230708 to -26.0378374769292	****	<0.0001
DMSO vs. 5 PE_F	-80	-84.9621625230708 to -75.0378374769292	****	<0.0001
DMSO vs. 10 PE_F	-98	-102.962162523071 to -93.0378374769292	****	<0.0001
DMSO vs. ACV	0	-4.96216252307083 to 4.96216252307083	ns	>0.9999
DMSO vs. Dex0.1	-100	-104.962162523071 to -95.0378374769292	****	<0.0001
DMSO vs. Dex0.2	-100	-104.962162523071 to -95.0378374769292	****	<0.0001
Effect of dried extracts against HSV-1_WT in pre-entry step				
DMSO vs. 0.5 LE_D	-25	-34.1521256101869 to -15.8478743898131	****	<0.0001
DMSO vs. 1 LE_D	-40.3333	-49.4854589435202 to -31.1812077231465	****	<0.0001
DMSO vs. 2.5 LE_D	-97.6667	-106.818792276854 to -88.5145410564798	****	<0.0001
DMSO vs. 5 LE_D	-95	-104.152125610187 to -85.8478743898131	****	<0.0001
DMSO vs. 10 LE_D	-99	-108.152125610187 to -89.8478743898131	****	<0.0001

DMSO vs. 0.5 HM_D	-22.3333	-31.4854589435202 to -13.1812077231465	****	<0.0001
DMSO vs. 1 HM_D	-35	-44.1521256101869 to -25.8478743898131	****	<0.0001
DMSO vs. 2.5 HM_D	-77.3333	-86.4854589435202 to -68.1812077231465	****	<0.0001
DMSO vs. 5 HM_D	-98	-107.152125610187 to -88.8478743898131	****	<0.0001
DMSO vs. 10 HM_D	-99	-108.152125610187 to -89.8478743898131	****	<0.0001
DMSO vs. 0.5 PE_D	-3.66667	-12.8187922768535 to 5.48545894352021	ns	0.916543
DMSO vs. 1 PE_D	-18.3333	-27.4854589435202 to -9.18120772314645	****	<0.0001
DMSO vs. 2.5 PE_D	-37.3333	-46.4854589435202 to -28.1812077231465	****	<0.0001
DMSO vs. 5 PE_D	-95	-104.152125610187 to -85.8478743898131	****	<0.0001
DMSO vs. 10 PE_D	-98.3333	-107.485458943520 to -89.1812077231465	****	<0.0001
DMSO vs. ACV	0	-9.15212561018688 to 9.15212561018688	ns	>0.9999
DMSO vs. Dex0.1	-100	-109.152125610187 to -90.8478743898131	****	<0.0001
DMSO vs. Dex0.2	-100	-109.152125610187 to -90.8478743898131	****	<0.0001
Effect of fresh extracts against HSV-1_dxpiii in pre-entry step				
DMSO vs. 0.5 LE_F	-26.19	-29.4841189856769 to -22.8958810143231	****	<0.0001
DMSO vs. 1 LE_F	-51.36	-54.6541189856769 to -48.0658810143231	****	<0.0001
DMSO vs. 2.5 LE_F	-84.01	-87.3041189856769 to -80.7158810143231	****	<0.0001
DMSO vs. 5 LE_F	-100	-103.294118985677 to -96.7058810143231	****	<0.0001
DMSO vs. 10 LE_F	-100	-103.294118985677 to -96.7058810143231	****	<0.0001
DMSO vs. 0.5 HM_F	-24.49	-27.7841189856769 to -21.1958810143231	****	<0.0001
DMSO vs. 1 HM_F	-51.02	-54.3141189856769 to -47.7258810143231	****	<0.0001
DMSO vs. 2.5 HM_F	-82.31	-85.6041189856769 to -79.0158810143231	****	<0.0001
DMSO vs. 5 HM_F	-100	-103.294118985677 to -96.7058810143231	****	<0.0001
DMSO vs. 10 HM_F	-100	-103.294118985677 to -96.7058810143231	****	<0.0001
DMSO vs. 0.5 PE_F	-20.07	-23.3641189856769 to -16.7758810143231	****	<0.0001
DMSO vs. 1 PE_F	-30.27	-33.5641189856769 to -26.9758810143231	****	<0.0001
DMSO vs. 2.5 PE_F	-53.74	-57.0341189856769 to -50.4458810143231	****	<0.0001
DMSO vs. 5 PE_F	-78.57	-81.8641189856769 to -75.2758810143231	****	<0.0001
DMSO vs. 10 PE_F	-100	-103.294118985677 to -96.7058810143231	****	<0.0001
DMSO vs. ACV	0	-3.29411898567691 to 3.29411898567691	ns	>0.9999
DMSO vs. Dex0.1	-100	-103.294118985677 to -96.7058810143231	****	<0.0001
DMSO vs. Dex0.2	-100	-103.294118985677 to -96.7058810143231	****	<0.0001
Effect of dried extracts against HSV-1_dxpiii in pre-entry step				
DMSO vs. 0.5 LE_D	-32.1533	-36.1427852345061 to -28.1638814321606	****	<0.0001
DMSO vs. 1 LE_D	-55.6867	-59.6761185678394 to -51.6972147654939	****	<0.0001
DMSO vs. 2.5 LE_D	-88.6267	-92.6161185678394 to -84.6372147654939	****	<0.0001
DMSO vs. 5 LE_D	-100	-103.989451901173 to -96.0105480988272	****	<0.0001
DMSO vs. 10 LE_D	-100	-103.989451901173 to -96.0105480988272	****	<0.0001
DMSO vs. 0.5 HM_D	-30.59	-34.5794519011728 to -26.6005480988272	****	<0.0001
DMSO vs. 1 HM_D	-53.3333	-57.3227852345061 to -49.3438814321606	****	<0.0001
DMSO vs. 2.5 HM_D	-85.1	-89.0894519011728 to -81.1105480988272	****	<0.0001
DMSO vs. 5 HM_D	-100	-103.989451901173 to -96.0105480988272	****	<0.0001
DMSO vs. 10 HM_D	-100	-103.989451901173 to -96.0105480988272	****	<0.0001
DMSO vs. 0.5 PE_D	-27.8433	-31.8327852345061 to -23.8538814321606	****	<0.0001
DMSO vs. 1 PE_D	-51.37	-55.3594519011728 to -47.3805480988272	****	<0.0001
DMSO vs. 2.5 PE_D	-62.7467	-66.7361185678394 to -58.7572147654939	****	<0.0001
DMSO vs. 5 PE_D	-76.4707	-80.4601185678394 to -72.4812147654939	****	<0.0001
DMSO vs. 10 PE_D	-100	-103.989451901173 to -96.0105480988272	****	<0.0001
DMSO vs. ACV	0	-3.98945190117276 to 3.98945190117276	ns	>0.9999
DMSO vs. Dex0.1	-100	-103.989451901173 to -96.0105480988272	****	<0.0001
DMSO vs. Dex0.2	-100	-103.989451901173 to -96.0105480988272	****	<0.0001
Effect of fresh extracts against HSV-1_WT in post-entry step				
DMSO vs. 0.5 LE_F	-17	-30.1229756712086 to -3.87702432879144	**	0.005241
DMSO vs. 1 LE_F	-30	-43.1229756712086 to -16.8770243287914	****	<0.0001
DMSO vs. 2.5 LE_F	-46.3333	-59.4563090045419 to -33.2103576621248	****	<0.0001
DMSO vs. 5 LE_F	-72.6667	-85.7896423378752 to -59.5436909954581	****	<0.0001
DMSO vs. 10 LE_F	-96.6667	-109.789642337875 to -83.5436909954581	****	<0.0001
DMSO vs. 0.5 HM_F	-8	-21.1229756712086 to 5.12297567120856	ns	0.478764
DMSO vs. 1 HM_F	-18.3333	-31.4563090045419 to -5.21035766212477	**	0.002278

DMSO vs. 2.5 HM_F	-43.6667	-56.7896423378752 to -30.5436909954581	****	<0.0001
DMSO vs. 5 HM_F	-57.3333	-70.4563090045419 to -44.2103576621248	****	<0.0001
DMSO vs. 10 HM_F	-82.3333	-95.4563090045419 to -69.2103576621248	****	<0.0001
DMSO vs. 0.5 PE_F	0	-13.1229756712086 to 13.1229756712086	ns	>0.9999
DMSO vs. 1 PE_F	-11.6667	-24.7896423378752 to 1.45630900454190	ns	0.105304
DMSO vs. 2.5 PE_F	-21.3333	-34.4563090045419 to -8.21035766212477	***	0.000344
DMSO vs. 5 PE_F	-43.3333	-56.4563090045419 to -30.2103576621248	****	<0.0001
DMSO vs. 10 PE_F	-59.6667	-72.7896423378752 to -46.5436909954581	****	<0.0001
DMSO vs. ACV	-98.3333	-111.456309004542 to -85.2103576621248	****	<0.0001
Effect of dried extracts against HSV-1_WT in post-entry step				
DMSO vs. 0.5 LE_D	-23.3333	-32.6987329206689 to -13.9679337459978	****	<0.0001
DMSO vs. 1 LE_D	-47.3333	-56.6987329206689 to -37.9679337459978	****	<0.0001
DMSO vs. 2.5 LE_D	-63.3333	-72.6987329206689 to -53.9679337459978	****	<0.0001
DMSO vs. 5 LE_D	-81.3333	-90.6987329206689 to -71.9679337459978	****	<0.0001
DMSO vs. 10 LE_D	-96	-105.365399587336 to -86.6346004126645	****	<0.0001
DMSO vs. 0.5 HM_D	-14.6667	-24.0320662540022 to -5.30126707933113	***	0.000546
DMSO vs. 1 HM_D	-46.6667	-56.0320662540022 to -37.3012670793311	****	<0.0001
DMSO vs. 2.5 HM_D	-52.6667	-62.0320662540022 to -43.3012670793311	****	<0.0001
DMSO vs. 5 HM_D	-69.6667	-79.0320662540022 to -60.3012670793311	****	<0.0001
DMSO vs. 10 HM_D	-90.6667	-100.032066254002 to -81.3012670793311	****	<0.0001
DMSO vs. 0.5 PE_D	-5.66667	-15.0320662540022 to 3.69873292066887	ns	0.48804
DMSO vs. 1 PE_D	-19.6667	-29.0320662540022 to -10.3012670793311	****	<0.0001
DMSO vs. 2.5 PE_D	-33.6667	-43.0320662540022 to -24.3012670793311	****	<0.0001
DMSO vs. 5 PE_D	-58.3333	-67.6987329206689 to -48.9679337459978	****	<0.0001
DMSO vs. 10 PE_D	-86.6667	-96.0320662540022 to -77.3012670793311	****	<0.0001
DMSO vs. ACV	-99.6667	-109.032066254002 to -90.3012670793311	****	<0.0001
Effect of fresh extracts against HSV-1_dxpiii in post-entry step				
DMSO vs. 0.5 LE_F	-3.66667	-11.7295763321870 to 4.39624299885370	ns	0.809386
DMSO vs. 1 LE_F	-32	-40.0629096655204 to -23.9370903344796	****	<0.0001
DMSO vs. 2.5 LE_F	-53.6667	-61.7295763321870 to -45.6037570011463	****	<0.0001
DMSO vs. 5 LE_F	-83	-91.0629096655204 to -74.9370903344796	****	<0.0001
DMSO vs. 10 LE_F	-95	-103.062909665520 to -86.9370903344796	****	<0.0001
DMSO vs. 0.5 HM_F	-0.66667	-8.72957633218703 to 7.39624299885370	ns	0.999686
DMSO vs. 1 HM_F	-28.6667	-36.7295763321870 to -20.6037570011463	****	<0.0001
DMSO vs. 2.5 HM_F	-44	-52.0629096655204 to -35.9370903344796	****	<0.0001
DMSO vs. 5 HM_F	-73	-81.0629096655204 to -64.9370903344796	****	<0.0001
DMSO vs. 10 HM_F	-93	-101.062909665520 to -84.9370903344796	****	<0.0001
DMSO vs. 0.5 PE_F	0	-8.06290966552036 to 8.06290966552036	ns	>0.9999
DMSO vs. 1 PE_F	-9	-17.0629096655204 to -0.937090334479636	*	0.021489
DMSO vs. 2.5 PE_F	-16.6667	-24.7295763321870 to -8.60375700114630	****	<0.0001
DMSO vs. 5 PE_F	-47.3333	-55.3962429988537 to -39.2704236678130	****	<0.0001
DMSO vs. 10 PE_F	-79.3333	-87.3962429988537 to -71.2704236678130	****	<0.0001
DMSO vs. ACV	-4	-12.0629096655204 to 4.06290966552036	ns	0.724809
Effect of dried extracts against HSV-1_dxpiii in post-entry step				
DMSO vs. 0.5 LE_D	-7	-13.9720386334302 to -0.0279613665697873	*	0.048613
DMSO vs. 1 LE_D	-20	-26.9720386334302 to -13.0279613665698	****	<0.0001
DMSO vs. 2.5 LE_D	-58.6667	-65.6387053000969 to -51.6946280332365	****	<0.0001
DMSO vs. 5 LE_D	-87.6667	-94.6387053000969 to -80.6946280332365	****	<0.0001
DMSO vs. 10 LE_D	-100	-106.972038633430 to -93.0279613665698	****	<0.0001
DMSO vs. 0.5 HM_D	-2	-8.97203863343021 to 4.97203863343021	ns	0.989496
DMSO vs. 1 HM_D	-20	-26.9720386334302 to -13.0279613665698	****	<0.0001
DMSO vs. 2.5 HM_D	-51.3333	-58.3053719667635 to -44.3612946999031	****	<0.0001
DMSO vs. 5 HM_D	-79.3333	-86.3053719667635 to -72.3612946999031	****	<0.0001
DMSO vs. 10 HM_D	-100	-106.972038633430 to -93.0279613665698	****	<0.0001
DMSO vs. 0.5 PE_D	0	-6.97203863343021 to 6.97203863343021	ns	>0.9999
DMSO vs. 1 PE_D	-12.3333	-19.3053719667635 to -5.36129469990312	***	0.000101
DMSO vs. 2.5 PE_D	-49.6667	-56.6387053000969 to -42.6946280332365	****	<0.0001
DMSO vs. 5 PE_D	-69	-75.9720386334302 to -62.0279613665698	****	<0.0001
DMSO vs. 10 PE_D	-98	-104.972038633430 to -91.0279613665698	****	<0.0001
DMSO vs. ACV	-4.33333	-11.3053719667635 to 2.63870530009688	ns	0.454843
HSV-1 *UL30* copy number in HSV-1_WT-infected Vero cells				

DMSO vs. LE_F	199233.3	162819.074315100 to 235647.592351566	****	<0.0001
DMSO vs. HM_F	199233.3	162819.074315100 to 235647.592351566	****	<0.0001
DMSO vs. PE_F	198483.3	162069.074315100 to 234897.592351566	****	<0.0001
DMSO vs. LE_D	200550	164135.740981767 to 236964.259018233	****	<0.0001
DMSO vs. HM_D	200300	163885.740981767 to 236714.259018233	****	<0.0001
DMSO vs. PE_D	198850	162435.740981767 to 235264.259018233	****	<0.0001
DMSO vs. ACV	200426.7	164012.407648434 to 236840.925684900	****	<0.0001
US6 mRNA expression level in HSV-1_WT-infected Vero cells				
DMSO vs. LE_F	0.828362	0.762551120472755 to 0.894172146193911	****	<0.0001
DMSO vs. HM_F	0.781369	0.715558853806089 to 0.847179879527245	****	<0.0001
DMSO vs. PE_F	0.656588	0.590777587139422 to 0.722398612860578	****	<0.0001
DMSO vs. LE_D	0.990453	0.924642453806088 to 1.05626347952724	****	<0.0001
DMSO vs. HM_D	0.990284	0.924473720472755 to 1.05609474619391	****	<0.0001
DMSO vs. PE_D	0.982801	0.916990153806089 to 1.04861117952724	****	<0.0001
DMSO vs. ACV	0.996552	0.930741720472755 to 1.06236274619391	****	<0.0001
HSV-1 *UL30* copy number in HSV-1_dxpiii-infected Vero cells				
DMSO vs. LE_F	307221.3	270521.206211214 to 343921.460455453	****	<0.0001
DMSO vs. HM_F	307746.7	271046.539544547 to 344446.793788786	****	<0.0001
DMSO vs. PE_F	267666.7	230966.539544547 to 304366.793788786	****	<0.0001
DMSO vs. LE_D	308213.3	271513.206211214 to 344913.460455453	****	<0.0001
DMSO vs. HM_D	308506	271805.872877880 to 345206.127122120	****	<0.0001
DMSO vs. PE_D	306200	269499.872877880 to 342900.127122120	****	<0.0001
DMSO vs. ACV	110000	73299.8728778804 to 146700.127122120	****	<0.0001
US6 mRNA expression level in HSV-1_dxpiii-infected Vero cells				
DMSO vs. LE_F	0.848439	0.577770746929619 to 1.11910725307038	****	<0.0001
DMSO vs. HM_F	0.829417	0.558748413596286 to 1.10008491973705	****	<0.0001
DMSO vs. PE_F	0.583102	0.312433746929619 to 0.853770253070381	****	<0.0001
DMSO vs. LE_D	0.910824	0.640155413596286 to 1.18149191973705	****	<0.0001
DMSO vs. HM_D	0.879944	0.609276080262952 to 1.15061258640371	****	<0.0001
DMSO vs. PE_D	0.749842	0.479173413596286 to 1.02050991973705	****	<0.0001
DMSO vs. ACV	0.113978	-0.156690586403714 to 0.384645919737047	ns	0.709431
NFKB1 mRNA expression level in HSV-1_WT-infected Vero cells				
DMSO vs. LE_D	0.7844	0.59581 to 0.97298	****	<0.0001
DMSO vs. HM_D	0.79576	0.60717 to 0.98434	****	<0.0001
DMSO vs. PE_D	0.57626	0.38768 to 0.76484	***	0.000202
DMSO vs. ACV	0.88458	0.69600 to 1.0732	****	<0.0001
IL6 mRNA expression level in HSV-1_WT-infected Vero cells				
DMSO vs. LE_D	-4.386	-7.497 to -1.275	**	0.007407
DMSO vs. HM_D	-2.094	-5.205 to 1.017	ns	0.222956
DMSO vs. PE_D	-1.845	-4.956 to 1.266	ns	0.311195
DMSO vs. ACV	-10.9	-14.01 to -7.789	****	<0.0001
STAT3 mRNA expression level in HSV-1_WT-infected Vero cells				
DMSO vs. LE_D	-0.7197	-1.603 to 0.1635	ns	0.118668
DMSO vs. HM_D	-0.07625	-0.9594 to 0.8069	ns	0.99694
DMSO vs. PE_D	-1.758	-2.641 to -0.8744	***	0.000644
DMSO vs. ACV	0.22	-0.6632 to 1.103	ns	0.879014
IL6 mRNA expression level in HSV-1_WT-infected CaSki cells				
DMSO vs. LE_D	-2.573	-4.441 to -0.7060	**	0.0066
DMSO vs. HM_D	-0.004574	-1.872 to 1.863	ns	>0.9999
DMSO vs. PE_D	0.3766	-1.491 to 2.244	ns	0.9485
DMSO vs. ACV	-7.301	-9.169 to -5.434	****	<0.0001
IL6 mRNA expression level in HSV-1_dxpiii-infected CaSki cells				
DMSO vs. LE_D	-2.872	-4.051 to -1.693	****	<0.0001
DMSO vs. HM_D	0.2508	-0.9281 to 1.430	ns	0.9386
DMSO vs. PE_D	0.1926	-0.9863 to 1.372	ns	0.9749
DMSO vs. ACV	-1.006	-2.185 to 0.1731	ns	0.1052
STAT3 mRNA expression level in HSV-1_WT-infected CaSki cells				
DMSO vs. LE_D	-0.8300	-3.371 to 1.711	ns	0.7855
DMSO vs. HM_D	0.08305	-2.458 to 2.624	ns	0.9999
DMSO vs. PE_D	-1.564	-4.105 to 0.9774	ns	0.3096

DMSO vs. ACV	0.03964	-2.502 to 2.581	ns	>0.9999
STAT3 mRNA expression level in HSV-1_dxpiii-infected CaSki cells				
DMSO vs. LE_D	0.2953	-0.4297 to 1.020	ns	0.6428
DMSO vs. HM_D	0.2494	-0.4756 to 0.9744	ns	0.7559
DMSO vs. PE_D	-0.6078	-1.333 to 0.1172	ns	0.1131
DMSO vs. ACV	0.1421	-0.5829 to 0.8671	ns	0.9530
DMSO vs. LE_D	0.4975	-0.1003 to 1.095	ns	0.1165
DMSO vs. HM_D	0.4338	-0.1639 to 1.032	ns	0.1922
DMSO vs. PE_D	0.2145	-0.3832 to 0.8122	ns	0.7301
DMSO vs. ACV	0.7939	0.1961 to 1.392	**	0.0086
NFKB1 mRNA expression level in HSV-1_WT-infected CaSki cells				
DMSO vs. LE_D	0.4975	-0.1003 to 1.095	ns	0.1165
DMSO vs. HM_D	0.4338	-0.1639 to 1.032	ns	0.1922
DMSO vs. PE_D	0.2145	-0.3832 to 0.8122	ns	0.7301
DMSO vs. ACV	0.7939	0.1961 to 1.392	**	0.0086
NFKB1 mRNA expression level in HSV-1_dxpiii-infected CaSki cells				
DMSO vs. LE_D	0.7850	0.3907 to 1.179	***	0.0003
DMSO vs. HM_D	0.6041	0.2097 to 0.9984	**	0.0029
DMSO vs. PE_D	0.4524	0.05803 to 0.8467	*	0.0230
DMSO vs. ACV	0.5721	0.1778 to 0.9665	**	0.0044
UL30 mRNA expression level in HSV-1_WT-infected CaSki cells				
DMSO vs. LE_D	0.4227	-0.02299 to 0.8685	ns	0.0652
DMSO vs. HM_D	0.4942	0.04849 to 0.9399	*	0.0282
DMSO vs. PE_D	0.4573	0.01159 to 0.9030	*	0.0437
DMSO vs. ACV	0.6957	0.2500 to 1.141	**	0.0024
UL30 mRNA expression level in HSV-1_dxpiii-infected CaSki cells				
DMSO vs. LE_D	0.2758	-0.2309 to 0.7825	ns	0.4091
DMSO vs. HM_D	0.5779	0.07119 to 1.085	*	0.0238
DMSO vs. PE_D	0.5098	0.003133 to 1.017	*	0.0484
DMSO vs. ACV	0.3038	-0.2029 to 0.8105	ns	0.3302
US6 mRNA expression level in HSV-1_WT-infected CaSki cells				
DMSO vs. LE_D	0.7822	0.4038 to 1.161	***	0.0002
DMSO vs. HM_D	0.7079	0.3296 to 1.086	***	0.0005
DMSO vs. PE_D	0.6028	0.2245 to 0.9812	**	0.0020
DMSO vs. ACV	0.7622	0.3838 to 1.141	***	0.0002
US6 mRNA expression level in HSV-1_dxpiii-infected CaSki cells				
DMSO vs. LE_D	0.7850	0.1350 to 1.435	*	0.0165
DMSO vs. HM_D	0.6041	-0.04594 to 1.254	ns	0.0719
DMSO vs. PE_D	0.2321	-0.4179 to 0.8821	ns	0.7331
DMSO vs. ACV	0.1442	-0.5058 to 0.7942	ns	0.9296
IL6 mRNA expression level in uninfected CaSki cells				
DMSO vs. LE_D	0.9363	0.6497 to 1.223	****	<0.0001
DMSO vs. HM_D	0.2947	0.008138 to 0.5812	*	0.0431
DMSO vs. PE_D	0.2002	-0.08636 to 0.4867	ns	0.2171
DMSO vs. ACV	0.05095	-0.2356 to 0.3375	ns	0.9662
IL6 mRNA expression level in uninfected Vero cells				
DMSO vs. LE_D	0.4962	0.2386 to 0.7539	***	0.0004
DMSO vs. HM_D	0.1404	-0.1172 to 0.3980	ns	0.4080
DMSO vs. PE_D	0.4020	0.1444 to 0.6596	**	0.0024
DMSO vs. ACV	0.6205	0.3629 to 0.8782	****	<0.0001
STAT3 mRNA expression level in uninfected CaSki cells				
DMSO vs. LE_D	0.9903	0.7530 to 1.228	****	<0.0001
DMSO vs. HM_D	0.7936	0.5563 to 1.031	****	<0.0001
DMSO vs. PE_D	0.8259	0.5886 to 1.063	****	<0.0001
DMSO vs. ACV	0.6906	0.4533 to 0.9279	****	<0.0001
STAT3 mRNA expression level in uninfected Vero cells				
DMSO vs. LE_D	0.7254	0.5264 to 0.9244	****	<0.0001
DMSO vs. HM_D	0.8090	0.6100 to 1.008	****	<0.0001
DMSO vs. PE_D	0.2361	0.03706 to 0.4351	*	0.0173
DMSO vs. ACV	-0.002411	-0.2014 to 0.1966	ns	>0.9999

NFKB1 mRNA expression level in uninfected CaSki cells				
DMSO vs. LE_D	0.7128	-0.1411 to 1.567	ns	0.1151
DMSO vs. HM_D	0.3158	-0.5381 to 1.170	ns	0.7104
DMSO vs. PE_D	0.7626	-0.09127 to 1.616	ns	0.0863
DMSO vs. ACV	0.6324	-0.2214 to 1.486	ns	0.1796
NFKB1 mRNA expression level in uninfected Vero cells				
DMSO vs. LE_D	-1.187	-3.722 to 1.349	ns	0.5781
DMSO vs. HM_D	0.3353	-2.200 to 2.871	ns	0.9916
DMSO vs. PE_D	0.3420	-2.194 to 2.878	ns	0.9910
DMSO vs. ACV	-4.136	-6.671 to -1.600	**	0.0012
